# Structural Studies of GABA_A_ Receptor Binding Sites: Which Experimental Structure Tells us What?

**DOI:** 10.3389/fnmol.2016.00044

**Published:** 2016-06-16

**Authors:** Roshan Puthenkalam, Marcel Hieckel, Xenia Simeone, Chonticha Suwattanasophon, Roman V. Feldbauer, Gerhard F. Ecker, Margot Ernst

**Affiliations:** ^1^Department of Molecular Neurosciences, Medical University of ViennaVienna, Austria; ^2^Department of Pharmaceutical Chemistry, University of ViennaVienna, Austria; ^3^Austrian Research Institute for Artificial Intelligence (OFAI)Vienna, Austria

**Keywords:** GABA_A_ receptors, subtype selectivity, binding pockets, allosteric modulatory sites, conformations

## Abstract

Atomic resolution structures of cys-loop receptors, including one of a γ-aminobutyric acid type A receptor (GABA_A_ receptor) subtype, allow amazing insights into the structural features and conformational changes that these pentameric ligand-gated ion channels (pLGICs) display. Here we present a comprehensive analysis of more than 30 cys-loop receptor structures of homologous proteins that revealed several allosteric binding sites not previously described in GABA_A_ receptors. These novel binding sites were examined in GABA_A_ receptor homology models and assessed as putative candidate sites for allosteric ligands. Four so far undescribed putative ligand binding sites were proposed for follow up studies based on their presence in the GABA_A_ receptor homology models. A comprehensive analysis of conserved structural features in GABA_A_ and glycine receptors (GlyRs), the glutamate gated ion channel, the bacterial homologs *Erwinia chrysanthemi* (ELIC) and *Gloeobacter violaceus* GLIC, and the serotonin type 3 (5-HT_3_) receptor was performed. The conserved features were integrated into a master alignment that led to improved homology models. The large fragment of the intracellular domain that is present in the structure of the 5-HT_3_ receptor was utilized to generate GABA_A_ receptor models with a corresponding intracellular domain fragment. Results of mutational and photoaffinity ligand studies in GABA_A_ receptors were analyzed in the light of the model structures. This led to an assignment of candidate ligands to two proposed novel pockets, candidate binding sites for furosemide and neurosteroids in the trans-membrane domain were identified. The homology models can serve as hypotheses generators, and some previously controversial structural interpretations of biochemical data can be resolved in the light of the presented multi-template approach to comparative modeling. Crystal and cryo-EM microscopic structures of the closest homologs that were solved in different conformational states provided important insights into structural rearrangements of binding sites during conformational transitions. The impact of structural variation and conformational motion on the shape of the investigated binding sites was analyzed. Rules for best template and alignment choice were obtained and can generally be applied to modeling of cys-loop receptors. Overall, we provide an updated structure based view of ligand binding sites present in GABA_A_ receptors.

## Introduction

γ-aminobutyric acid type A receptors (GABA_A_ receptors) are important drug targets in the treatment of various neuropsychiatric conditions (Sieghart, 2015), and for the induction and maintenance of general anesthesia (Olsen et al., 2013; Antkowiak, 2015). Clinically used compounds such as benzodiazepines, etomidate, propofol and others possess allosteric binding sites and the binding to these sites changes the response of the receptors to the agonist GABA. These pentameric receptors are assembled in mammalian species as homo- or heteropentamers from a repertoire of 19 subunits (Sieghart, 1995; Olsen and Sieghart, 2008). This results in a large variety of receptor subtypes (Olsen and Sieghart, 2008) that display distinctive properties. Structurally seen, GABA_A_ receptors are typical members of the superfamily of pentameric ligand-gated ion channels (pLGICs). Other members of this superfamily, which are also known as cys-loop receptors, are the nicotinic acetylcholine receptors (nAChRs), the serotonin type 3 receptors (5-HT_3_Rs) and the glycine receptors (GlyRs; Unwin, 2005; Du et al., 2015; Ernst and Sieghart, 2015; Huang et al., 2015).

Most GABA_A_ receptors are heteropentamers, whereby the most abundant receptors’ subunit composition is α1, β2 and γ2 subunits in the ratio 2:2:1 (Olsen and Sieghart, 2008). For the interaction of drugs or other ligands with these receptors, the interfaces between subunits play an important role as they harbor binding sites. However, only few interfaces have been studied extensively and confirmed to exist (Duncalfe et al., 1996; Smith and Olsen, 2000; Chiara et al., 2012, 2013; Jayakar et al., 2014). Receptors composed of α1, β3 and γ2 subunits were shown to be arranged as β3−α1−γ2−β3−α1 (Tretter et al., 1997, see “Supplementary Figure 1”), and in experiments with concatenated subunits the same arrangement was identified for α1β2γ2 receptors (Baumann et al., 2001). The counter-clockwise geometry (as viewed from extracellular) was determined later with a homology model based on the X-ray structure of the homologous acetylcholine binding protein (AChBP; Brejc et al., 2001; Ernst et al., 2003). The existing interfaces in these subtypes are thus β+/α1−, α1+/β−, α1+/γ2− and γ2+/β− (Tretter et al., 1997; Baumann et al., 2001). It is assumed that in αβγ subtypes with other α, β or γ isoforms the arrangement is the same.

Receptors composed of four and even five different subunits might also be formed, such as αβγ receptors containing two different α or β subunits (Verdoorn, 1994; Olsen and Sieghart, 2008). Examples for such subtypes are α1α3βγ receptors (Ralvenius et al., 2015) or α1α5− containing receptors (Araujo et al., 1999).

The arrangement of receptors that contain the δ subunit together with α and β subunits (αβδ subtypes) is less clear at this time (see “Supplementary Figure 1”). Atomic force microscopy identified in α4β3δ receptors the arrangement β3−α4−δ−β3−α4 (Barrera et al., 2008; Eaton et al., 2014). In this arrangement, δ is located in the same position as γ in the αβγ subtypes. For other δ− containing receptors also the arrangements β−α−δ−α−β or β−α−β−δ−α have been proposed (Kaur et al., 2009; Sigel et al., 2009). Thus, the interfaces α+/δ− or β+/δ−, as well as δ+/α− and δ+/β− could exist in principle (see “Supplementary Figure 1”). Lee et al. (2016) have demonstrated the existence of a GABA site at the β+/δ interface.

The most prominent receptors comprising only two different subunits are the αβ receptors in which the γ subunit is replaced by a β subunit (Baumann et al., 2001; Mortensen and Smart, 2006). Here, the β+/β− interface exists as well (Tretter et al., 1997; Baumann et al., 2001). Homopentameric GABA_A_ receptors can be formed of five ρ subunits. Other homopentamers and thus homo-interfaces possibly exist as well. Two different ρ subunits can assemble to form heteropentameric ρ receptors (Olsen and Sieghart, 2008). Additional ρ− containing interfaces may exist due to the identified co-assembly of ρ subunits with the γ2 subunit (Qian and Pan, 2002). The composition, stoichiometry and arrangement of receptors that contain the ε, θ and π subunits are so far unknown.

Of interest here are the localizations of ligand binding sites on a prototypical GABA_A_ receptor pentamer—independent of the identity of the subunits that form it. Subtype differences can then be studied in a next step, after binding site localization is established. Binding site localizations based on indirect methods and those based on atomic resolution structure determination are briefly reviewed. Structural data has been selected based on the presence of small molecule ligands in orthosteric and allosteric binding sites, for more historical reviews of cys-loop receptor structures see for example Lemoine et al. (2012), Nys et al. (2013), daCosta and Baenziger (2013), Lynagh and Pless (2014) and Sauguet et al. (2015).

**Figure 1:**
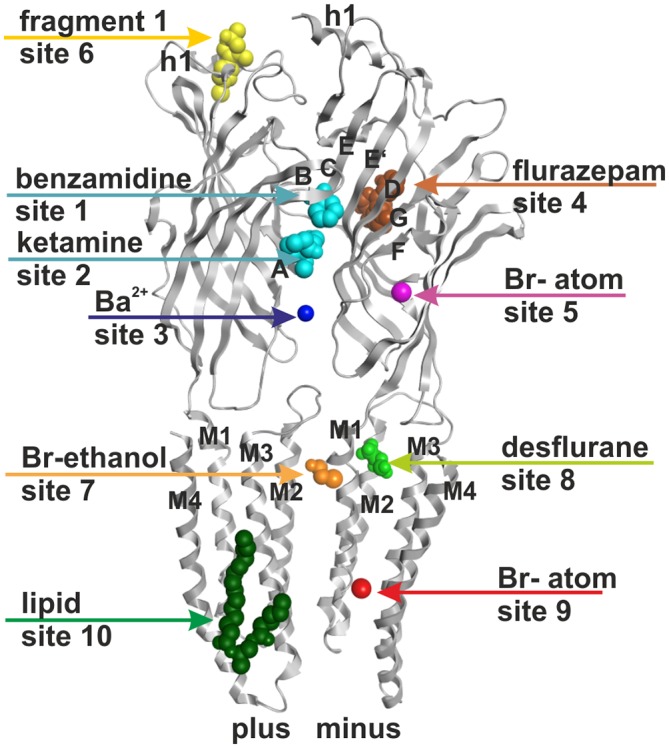
**Ten small ligand binding sites are found in atomic structures of γ-aminobutyric acid type A receptor (GABA_A_ receptor) homologs.** The figure shows a side view of a superposition of the protein data bank (PDB) files shown in boldface in Table 1. Representative ligand positions were chosen for display of the 10 studied sites. Two subunits of 4COF are shown in ribbon representation (gray). The ligands are depicted in space-filling representation, and the orginal PDB files are specified in Table 1. More examples of ligands in these 10 site types are given in Table 1 and “Supplementary Figure 2”.

Binding site localization of agonists and allosteric modulators have long been established to exist at specific extracellular interfaces, such as for the agonist GABA at the orthosteric sites such as β+/α− interfaces (Smith and Olsen, 1994) as well as allosteric sites for benzodiazepines at α+/γ− (Sigel and Lüscher, 2011) and for pyrazoloquinolinones at α+/β− interfaces (Sieghart et al., 2012). A Zn^2+^ site was proposed in the α+/β− interface in a unique position that does not overlap with the pyrazoloquinolinone site and is localized closer to the membrane (Hosie et al., 2003). Many structures of cys-loop receptor family members already exist with ligands bound to the extracellular domain (ECD) interface (for example Hansen et al., 2005; Hibbs and Gouaux, 2011; Pan et al., 2012a; Zimmermann et al., 2012; Miller and Aricescu, 2014; Du et al., 2015; Huang et al., 2015; Spurny et al., 2015) including a benzamidine bound crystal structure of a β3− homopentameric GABA_A_ receptor (Miller and Aricescu, 2014). Interestingly, three (sub-) sites are observed at the ECD-interface in the atomic structures (Pan et al., 2012a; Spurny et al., 2012, 2015; Zimmermann et al., 2012; Miller and Aricescu, 2014; Du et al., 2015; see Figure 1 and “Supplementary Figure 2”). The ECD-interface is largely formed by the so-called loops A-C from the principal (plus) subunit and D-G of the complementary (minus) subunit (Galzi and Changeux, 1994; Sigel and Buhr, 1997; Ernst et al., 2003). Additionally, cation binding sites have been observed in a localization closer to the trans-membrane domain (TMD) and outside of the region covered by loops A-G (Zimmermann et al., 2012).

The interfaces between subunits of GABA_A_ receptors also have been shown to contain specific binding sites in the TMD (TMD-interfaces). Highly efficient photoaffinity ligands allowed recently to assign specific TMD-interfaces to etomidate (β+/α−, Li et al., 2006; Chiara et al., 2012) and to barbiturate ligands (α+/β− and γ+/β−, Chiara et al., 2013). More complex results were obtained with photoreactive derivatives of propofol and in mutational studies. These seem to indicate that propofol sites exist in at least four different interfaces (Yip et al., 2013; Jayakar et al., 2014; Lynagh and Laube, 2014; Franks, 2015; Stern and Forman, 2016) and possibly at additional sites (Moraga-Cid et al., 2011)*.* As was reported recently, the α+/β− and γ+/β− containing interfaces in GABA_A_ receptors can also bind avermectin (Estrada-Mondragon and Lynch, 2015), while the β+ containing interfaces cannot accommodate this ligand. The action of many other ligands has tentatively been connected with usage of these pockets as well (Wingrove et al., 1994; Walters et al., 2000; McCracken et al., 2010; Hanrahan et al., 2015; Luger et al., 2015; Middendorp et al., 2015)*.* While the X-ray crystallographic structure of the β3− homopentameric GABA_A_ receptor (Miller and Aricescu, 2014) has no ligand bound at the TMD-interface, structures of several related proteins were determined with different ligands in positions consistent with the proposed binding sites for etomidate, barbiturates, avermectin and other ligands (for example Hibbs and Gouaux, 2011; Sauguet et al., 2013; Althoff et al., 2014; Du et al., 2015). These ligand bound structures, together with atomic structures in different conformational states (Althoff et al., 2014; Sauguet et al., 2014; Du et al., 2015), allow to investigate the structural properties of the ligand binding sites localized at TMD-interfaces.

The first allosteric binding site that was proposed to be localized in GABA_A_ receptors in a non-interface position is a recently described site used by endocannabinoids such as 2-arachidonglycerol (2-AG) and anandamide (Sigel et al., 2011). A crystal structure was found that contains a lipid molecule in the site that corresponds to the proposed 2-AG binding site (Bocquet et al., 2009), which therefore allows structural studies for this site in an occupied state.

A picrotoxinin binding site in the channel pore has been known to exist in GABA_A_ receptors, and was also observed in crystal structures (Curtis et al., 1969; Hibbs and Gouaux, 2011). Furthermore, additional ligands have been observed to bind in the ion pore of homologous proteins as well (Hilf et al., 2010; Spurny et al., 2013; Chen et al., 2015). Multiple additional localizations were observed for small molecule ligands in diverse structures (for example Bocquet et al., 2009; Nury et al., 2011; Pan et al., 2012a; Spurny et al., 2012, 2013, 2015; Zimmermann et al., 2012; Sauguet et al., 2013). Here we investigate sites that potentially can be targets of small molecule allosteric modulators of GABA_A_ receptor subtypes, revisit structural insight on the known allosteric ECD-interface and TMD-interface sites, and examine novel putative sites. All binding sites that were studied in the selected experimentally determined structures are summarized in Figure 1 and Table 1. Channel blockers that bind to the ion pore are not within the scope of this study which focuses on allosteric modulatory sites.

**Table 1:** **Crystal structures used as templates or for mapping of putative binding sites^1^**.

Pocket	PDB ID	Protein	Ligand	Reference
**Extracellular interface sites:**
ECD-interface (1)	**4COF**	GABA_A_ β3	Benzamidine (1, **cyan**)	Miller and Aricescu (2014)
ECD-interface (1)	3RIF	GluCl	Glutamate	Hibbs and Gouaux (2011)
ECD-interface (1)	3JAD	GlyR α1	Strychnine	Du et al. (2015)
ECD-interface (1)	3JAE	GlyR α1	Glycine	Du et al. (2015)
ECD-interface (1)	5CFB	GlyR α3	Strychnine	Huang et al. (2015)
ECD-interface (1)	2BYR	AChBP	Methyllycaconitine	Hansen et al. (2005)
ECD-interface (1)	2BYS	AChBP	Lobeline	Hansen et al. (2005)
ECD-interface (1)	4BFQ	AChBP	VUF9432	Stornaiuolo et al. (2013)
ECD-interface (1)	5AFJ	nAChR α7-AChBP	Lobeline	Spurny et al. (2015)
ECD-interface (1)	4A97	ELIC	R-Zopiclone	Spurny et al. (2012)
ECD-interface (1)	4A98	ELIC	Br-flurazepam	Spurny et al. (2012)
ECD-interface (2)	**4F8H**	GLIC	R-Ketamine (2, **cyan**)	Pan et al. (2012a)
ECD-interface (2)	**5AFJ**	nAChR α7-AChBP	Fragment 1	Spurny et al. (2015)
ECD-interface (3)	**2YN6**	ELIC	Ba2^+^ (3, **blue**)	Zimmermann et al. (2012)
**Extracellular sites not at interfaces:**
ECD-vestibule (4)	**2YOE**	ELIC	Flurazepam (4, **brown**)	Spurny et al. (2012)
ECD-vestibule (4)	**2YN6**	ELIC	Ba2^+^	Zimmermann et al. (2012)
ECD-vestibule (4)	**5AFM**	nAChR α7-AChBP	Fragment 4	Spurny et al. (2015)
ECD-helix 1 (6)	**5AFJ**	nAChR α7-AChBP	Fragment 1 (6, **yellow**)	Spurny et al. (2015)
ECD-core (5)	**3ZKR**	ELIC	Br-(omoform) (5, **magenta**)	Spurny et al. (2013)
**Trans-membrane interface sites:**
TMD-interface (7)	**4HFD**	GLIC (F14’A	Bromoform	Sauguet et al. (2013)
TMD-interface (7)	**4HFC**	GLIC (F14’A)	Br-ethanol (7, **orange**)	Sauguet et al. (2013)
TMD-interface (7)	**4HFE**	GLIC (F14’A)	Ethanol	Sauguet et al. (2013)
TMD-interface (7)	**3RIF**	GluCl	Avermectin	Hibbs and Gouaux (2011)
TMD-interface (7)	3RI5	GluCl	Avermectin	Hibbs and Gouaux (2011)
TMD-interface (7)	3RIA	GluCl	Avermectin	Hibbs and Gouaux (2011)
TMD-interface (7)	3RHW	GluCl	Avermectin	Hibbs and Gouaux (2011)
TMD-interface (7)	**4TNW**	GluCl	POPC	Althoff et al. (2014)
TMD-interface (7)	3JAF	GlyR α1	Avermectin	Du et al. (2015)
**Trans-membrane sites not at interfaces:**
TMD intra-subunit (8)	**3P4W**	GLIC	Desflurane (8, **green**)	Nury et al. (2011)
TMD intra-subunit (8)	**3P50**	GLIC	Propofol	Nury et al. (2011)
TMD intra-subunit (8)	**4HFD**	GLIC (F14’A)	Bromoform	Sauguet et al. (2013)
TMD intra-subunit (8)	**4HFH**	GLIC	Bromoform	Sauguet et al. (2013)
TMD M1/M4 (9)	**3ZKR**	ELIC	Br-(omoform; 9, **red**)	Spurny et al. (2013)
TMD M3/M4 (10)	**3EAM**	GLIC	Lipid (10, **dark green)**	Bocquet et al. (2009)
**Additional structures:**
	4PIR	5-HT_3A_		Hassaine et al. (2014)
	4TNV	GluCl		Althoff et al. (2014)
	2QC1	nAChR		Dellisanti et al. (2007)

*^1^Several PDB identifiers are listed multiple times because they contain more than one ligand, in different sites. Some structures contain additional ligands that are not listed in the table, the reader is referred to the original PDB records for complete lists of ligands*.

In several of the analyzed structures multiple ligands are present in diverse combinations. Some structures were determined successfully with agonist molecules in the ECD and allosteric modulators bound in TMD-sites (such as 3RIF, see Table 1). In the ECD-interface in one instance multiple copies of a ligand were observed (Stornaiuolo et al., 2013), and another structure features simultaneous occupation of two (sub-) sites in the ECD-interface by different ligands (Spurny et al., 2015; see Table 1). Multiple copies of ligands also have been observed in the TMD-interface and intra-subunit regions (Sauguet et al., 2013, see “Supplementary Figure 2”).

For the binding site localizations 4, 5, 6, 8 and 9 (see Figure 1) that have been observed in structures of the bacterial homologs GLIC and ELIC or members of the nAChR family it is unclear if they exist in GABA_A_ receptors at all. We thus addressed the question how binding sites from these homologous proteins map onto the GABA_A_ receptor crystal structure, and onto homology models of other GABA_A_ receptor subtypes. Based on this mapping we evaluated the likelihood of their existence and attempted to assign ligands whose binding sites on GABA_A_ receptors have not yet been localized to any of these novel pockets.

Furthermore, since only the structure of a single conformation is available of an engineered GABA_A_ receptor (Miller and Aricescu, 2014) neither conformational variability nor structural variability within the family can be studied at this time on the basis of this crystal structure. However, structures of the GlyR, the glutamate gated ion channel (GluCl) and GLIC in different conformations exist. These reveal conformational changes that happen in response to different events, such as binding of ligands to different binding sites (Althoff et al., 2014; Sauguet et al., 2014; Du et al., 2015). Thus, we employed multiple structures (Hibbs and Gouaux, 2011; Althoff et al., 2014; Du et al., 2015; Huang et al., 2015) in addition to the GABA_A_ receptor crystal structure (Miller and Aricescu, 2014) as templates to gain more insight into the impact of protein motion on pocket structures (see Table 1). We also studied the recently solved structure of the 5-HT_3_ receptor with a large intact intracellular domain (ICD) fragment (Hassaine et al., 2014) to examine the putative ICD structure in GABA_A_ receptors.

## Materials and Methods

### Template Selection

Apo- and ligand-bound structures of wild-type and mutant homologous proteins of the GABA_A_ receptors were extracted from the protein data bank (PDB)^1^. The analyzed structures are under the following accession numbers: AChBP (2BYR, 2BYS), ELIC (2YOE, 3ZKR, 2YN6, 4A97, 4A98), GLIC (3EAM, 3P4W, 3P50, 4F8H, 4HFH, 4HFE, 4HFD, 4HFC), GluCl (3RIF, 3RHW, 3RI5, 3RIA, 4TNV, 4TNW), 5-HT_3A_ (4PIR), nAChR α7-AChBP-chimera (5AFJ, 5AFM), GlyR α1 (3JAD, 3JAE, 3JAF), GlyR α3 (5CFB) and the homopentameric GABA_A_ receptor β3 (4COF) which is the only available GABA_A_ receptor so far. All structures had sufficient resolution and quality for this work, in which mainly protein backbone structure impacted on the results.

### Alignment Generation

Different crystal structures were analyzed for conserved elements at the PDBeFold webserver^2^ which is a tool that generates structural alignments based on secondary structure matching (SSM, Krissinel and Henrick, 2004).

**Figure 2:**
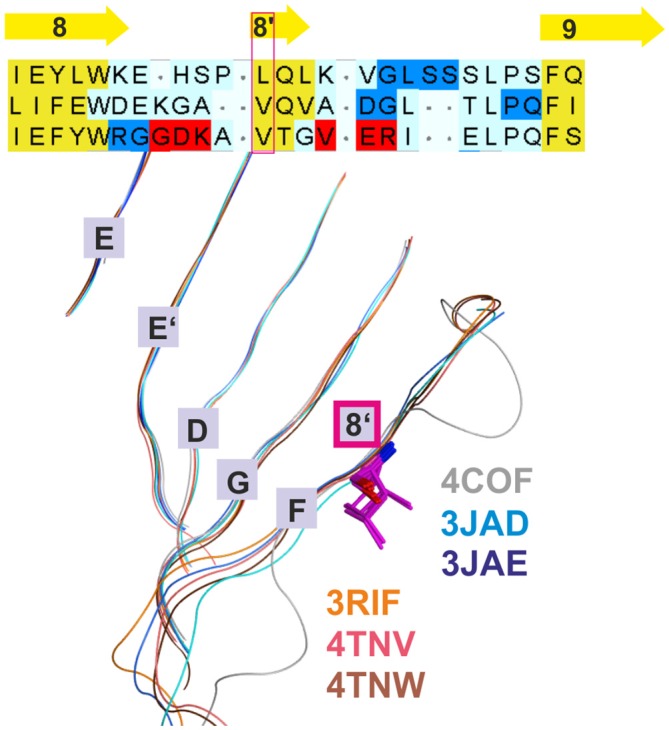
**Conserved strand 8’ limits alignment choices in loop F region.** The structural overlay of a single subunits’ extracellular domain (ECD) of all glycine receptor (GlyR) structures, all glutamate gated ion channel (GluCl) structures and the GABA_A_ receptor structure 4COF reveals a strict conservation of the short strand 8’, which always starts with a hydrophobic residue. The image shows the sequence alignment of the loop F region which results from the 3D superposition, and the perfect overlay of the hydrophobic residues in the indicated representative structures. Thus, for aligning sequences of subunits with unknown structure to the resolved structures, the hydrophobic position alignment that is emphasized by the magenta box must be enforced. “Supplementary Figure 3A” (the master alignment) shows a possible alignment of the 19 GABA_A_ receptor subunits in this region to the different structures to preserve strand 8’. Notably, *Gloeobacter violaceus* (GLIC) and *Erwinia chrysanthemi* (ELIC) also feature this conserved motif (see “Supplementary Figures 3D,E”).

GABA_A_ receptor sequences from the rat were obtained from the UniProt database^3^. The signal peptides of the sequences were removed, the sequences were trimmed at the N-terminal end and the ICD was replaced by a short linker (e.g., AGT for GluCl and GlyR based models and SQPARAA for 4COF based models). (Multi) sequence alignments of the GABA_A_ receptor sequences and the homologous proteins were generated with the ClustalX program^4^ (Thompson et al., 1994). PROMALS3D was used for the sequence-to-structure alignments (Pei et al., 2008). The results obtained in the 3D alignments were used to manually correct alignments obtained with ClustalX and PROMALS3D, see Figure 2 and “Supplementary Figure 3”. A master alignment for models based on 4COF as well as GlyR and GluCl structures is provided in “Supplementary Table 3A”, a PROMALS3D derived alignment of selected GABA_A_ receptor subunits with the 4PIR structure is provided in “Supplementary Table 3B”.

### Model Generation

**Figure 3:**
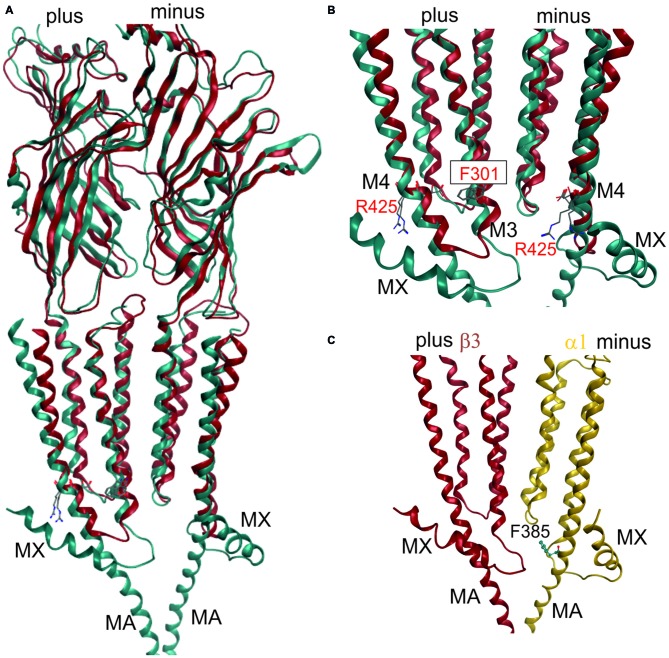
**3D superposition of 4PIR and 4COF and modeling of the GABA_A_ receptor intact intracellular domain (ICD). (A)** Superposition of 4PIR (cyan) and 4COF (red) shows high structural similarity between the distant homologs. **(B)** Magnified view of the trans-membrane domain (TMD)-ICD interface. Strictly conserved amino acids at the intracellular end of M3 and M4 superpose very well and are shown in stick representation, numbered according to 4COF (see also “Supplementary Figure 3B”). **(C)** Side view of a homology model of the β3+/α1− interface (α1: yellow, β3: red) of the GABA_A_ receptor with the ICD based on the 4PIR structure. α1F385 localizes to the α minus side of the interface in the model and has contacts with the β3+ pre-MX loop, and with the M1-M2 linker of α1. This residue that has been implicated in propofol modulatory action thus likely localizes to the interface forming part of the ICD.

The software MODELLER 9.9^5^ was used to generate homology models of the GABA_A_ receptors by using the satisfaction of spatial restraints method (Sali and Blundell, 1993). The needed inputs for MODELLER are: one or more PDB-file of the homologous template protein(s), an alignment of the template(s) and the (optionally) trimmed rat GABA_A_ receptor sequences, and a python script to run the process. Validation of the generated homology models was performed with the PROCHECK program and the protein geometry tool in MOE (Ramachandran-Plots, G-factor). Models based on 4COF as well as the GlyR and GluCl structures were conventional single template models based on the alignment shown in “Supplementary Figure 3A”. The model with an ICD fragment based on 4COF and 4PIR was based on 4COF for the ECD and the TMD, and on 4PIR in the ICD. The transition between the two templates was performed at the conserved M3 and M4 residues that are depicted in Figure 3. “Supplementary Figure 3” includes details and the alignment variant used for the Supplementary Model.

### Pocket Mapping

Structures of GLIC, ELIC, AChBP and the nAChR-AChBP chimera were superposed with 4COF at the PDBeFold webserver^6^ based on secondary structure matching (SSM, Krissinel and Henrick, 2004). Subsequently, the region around the ligands that were co-crystallized in different structures of GLIC, ELIC, AChBP and the nAChR-AChBP chimera respectively was mapped according to the SSM superposition as indicated in the alignments shown in “Supplementary Figures 3C–E” and processed as indicated in “Supplementary Figure 3” (workflow diagram). Pocket forming amino acids that were identified as putatively conserved among the parent structure and the GABA_A_ receptors were then highlighted in “Supplementary Figure 3A”.

### Analysis of Conformational Changes

To assess global changes to individual domains, subunits and at interfaces, RMSDs among α-carbon groups of appropriate superpositions were employed. Thus, whole single subunits and whole pentamers were superposed with the secondary structure matching algorithm (Krissinel and Henrick, 2004), and with a minimum RMSD calculator for subunit domains. Distances between α-carbons of pocket forming residues were measured and compared between the different models to study differences in pocket structures.

## Results

### GABA_A_ Receptor Homology Models Based on 4COF and Additional Templates

For many homology modeling studies concerned with GABA_A_ receptor subtypes, the obvious and best template will be the β3 GABA_A_ receptor structure (PDB ID 4COF, an engineered human β3 homopentamer lacking the ICD) itself. A source of uncertainty in 4COF based models comes from the variable sequence length among GABA_A_ receptor subunits, such as the longer F loop region in α subunits compared to β3, or the longer C loop of the ρ subunits (“Supplementary Figure 3A”). Thus, if the 4COF structure is used as template for other GABA_A_ receptor subunits, such as the α, γ or δ subunits, stretches of sequence with different length lead to so-called “INDELs” (concatenation of INsertion and DELetion, an alignment stretch in which sequence length differs and thus leads to insertion or deletion of amino acids relative to the aligned protein). In turn, alignment algorithms (Thompson et al., 1994; Shi et al., 2001; Pei et al., 2008) generate controversial solutions—a phenomenon known as the “alignment problem” in homology modeling.

Additional templates can be used to gain more insights into conserved structural elements that are not detectable by sequence alignment tools and thus resolve ambiguous alignments in variable regions. Use of additional structures has already been applied elegantly in the past to better align target sequences with the GluCl structure (Bergmann et al., 2013). This approach was also employed here, and all presented models are based on the alignment that results from combining 3D superposition information from multiple structures (Krissinel and Henrick, 2004) with sequence-to-structure tools (Pei et al., 2008; see “Materials and Methods” Section and “Supplementary Figure 3A”). The superposition of multiple structures using secondary structure matching as implemented in the PDBeFold web service (Krissinel and Henrick, 2004) successfully aligns structurally conserved amino acid residues even in the absence of sequence similarity (see Figure 2). This restricts the possible placement of INDELs considerably. If multiple 3D structures are superposed, a conserved motif (for example VTG in the β3 subunit) that forms a short strand 8′ emerges (see Figure 2 and the alignment in “Supplementary Figure 3A”) with the* hydrophobic// × (any amino acid)// hydrophobic (or small)//* pattern. Of this motif, the first hydrophobic position is conserved even in the GLIC and ELIC proteins, indicating an important structural role. Models based on alignments in which polar side chains are placed into this position are likely to be wrong as this hydrophobic residue seems to be important for the structural integrity (hydrophobic packing). If an alignment of a hydrophobic amino acid of, for example, GABA_A_ receptor α subunits with β3V175 is enforced, the number of possible alignment variants of the F loop regions is reduced considerably.

Once the master alignment has been established, it was employed to generate models using the MODELLER program (Sali and Blundell, 1993) based on several template structures. Sequence similarity is often used as criterion for template selection. The more stringent criterion of structural overlap can be employed if structures are available that allow to quantify structural similarity between remote family members. Thus, the structural overlap was assessed between the GABA_A_ receptor and structures of the GlyR, GluCl, GLIC, ELIC, the 5-HT_3_R and members of the AChBP/nAChR family. Homology models were subsequently built only on the basis of 4COF and the eukaryotic homologs (GlyR, GluCl and the 5-HT_3_R) as described in the methods. Structures from GLIC, ELIC, AChBP and nAChRs were used only for 3D superposition based pocket mapping, i.e., the localization of putative novel binding pockets for small molecule ligands, while the pockets were then studied in homology models based on the above listed templates. To study the ICD, the 5-HT_3_R, that contains a large fragment of the ICD (4PIR, Hassaine et al., 2014) was employed in a multi-template approach together with 4COF, see “Materials and Methods” Section. Due to the surprisingly high structural and conformational similarity between the TMDs of the 5-HT_3_R structure (4PIR) and the GABA_A_ receptor structure (4COF) and a very good overlap of the ICD-near parts of M3 and M4 (see Figure 3) it was possible to construct the two-templates models, see Figure 3. This structure for the first time provides insight into the putative structure of the N-terminal end of this important, highly variable and regulatory domain.

The fragment C-terminal of M3 in the 5-HT_3_R consists of a loop and a short α helix (termed MX), followed by missing amino acids due to the proteolysis that was performed (Hassaine et al., 2014). The C-terminal end of the ICD features the MA helix that was also observed in the nAChR structure (Unwin, 2005). The question which of these elements may also be present in GABA_A_ receptor subunits can be answered only tentatively on the basis of sequence alignments and sequence-to-structure alignments that thread the GABA_A_ receptor subunits’ sequences onto the 4PIR ICD structure and sequence. Basically, no conserved motifs are detected near the putative MX region by multi-sequence alignments. If single subunits or small multi-sequence alignments such as the six α subunits are aligned to the 4PIR structure using the PROMALS3D web service (Pei et al., 2008), the connecting loop between M3 is predicted to be as long as, or slightly shorter than the one seen in the 4PIR structure (see “Supplementary Figure 3B”). The MX helix forming sequence aligns reasonably well with the GABA_A_ receptor β subunits, and less convincing with the α subunits. For the γ subunits, no reasonable alignment can be obtained. Thus, some GABA_A_ receptor subunits may contain a similar motif, while others may lack it—consistent with the diverse functional roles of the ICD. The MA helix and the homologous stretch of sequences in the GABA_A_ receptor subunits also lack any obvious conserved motif, so alignments are different and remain ambiguous. However, for the C-terminal 20 amino acids of the ICD until the beginning of M4 all sequence-to-structure algorithms consistently align most GABA_A_ receptor subunits with the MA helix of 4PIR, strongly suggesting that MA is also present in most GABA_A_ receptor subunits (see “Supplementary Figure 3B”).

This is of particular interest because in a recent work it was shown that α1F385 in the ICD has a major impact on propofol’s ability to modulate GABA_A_ receptors (Moraga-Cid et al., 2011). This raises the question if there might be an additional propofol binding site in the ICD that accounts for the modulatory effects of this ligand, and what the functional role of the binding sites in the TMD might be that have been observed by competition assays (Chiara et al., 2012, 2013) and by photolabeling (Yip et al., 2013; Jayakar et al., 2014). The mutational analysis pointing at the crucial role for α1F385 (Moraga-Cid et al., 2011) could have identified a binding site residue, or a non-local factor that is important to stabilize a particular conformational state. Mutations at very distant sites (specifically at the plus side of the etomidate/propofol site near the extracellular end of the TMD) can also disrupt allosteric modulation by propofol (Krasowski et al., 2001), which suggests that α1F385 is needed for the transduction of a conformational change, but is not necessarily part of a propofol binding site. However, this interpretation offers no explanation for the lack of effect the α1F385 mutation has on etomidate potentiation (Moraga-Cid et al., 2011). An alternative, and rather speculative interpretation would be that propofol binds silently in the pockets used by etomidate and barbiturates, and exerts its modulatory effect by binding in a pocket near α1F385. This hypothesis would consolidate the competitive action (Li et al., 2010; Chiara et al., 2012) and the photoincorporation of *ortho-*propofol diazirine at position β3H267 (Yip et al., 2013) by the assumption of a silent site in the TMD and a propofol specific modulatory site near α1F385 (Moraga-Cid et al., 2011). In a homology model built on the basis of the alignment suggested by the PROMALS3D server (Pei et al., 2008; see “Supplementary Figure 3B”), this amino acid localizes near the M1-M2 linker of the same subunit on the α1 minus side, and near the pre-MX loop of the neighboring subunit’s plus side at the β+/α− interface. This position could be equally consistent with a binding site or a transduction site. Interestingly, the plus face of the MA helix has been photolabeled in the nAChR α subunit by azietomidate (Chiara et al., 2009b) and by chlorpromazine (Chiara et al., 2009a), see “Supplementary Figure 3F”. Further experiments are needed to clarify the functional role of this region in mediating ligand effects, and homology models of the ICD in GABA_A_ receptor subunits can serve as useful tools to select candidate residues for further mutational analysis.

### Mapping and Assessment of Binding Sites in Models of GABA_A_ Receptors

#### ECD-Interface (Orthosteric Site and Homologous Allosteric Sites), Subsites 1 and 2 and the Cation Site 3

**Figure 4:**
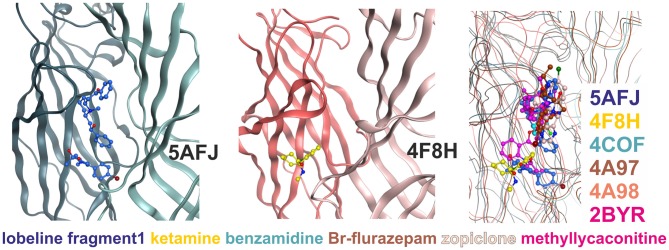
**Multiple ligand binding sites at the extracellular domain (ECD)- interface.** Left panel, 5AFJ: two different ligands occupy subsites 1 and 2 simultaneously in the nicotinic acetylcholine receptor (nAChR)- Acetylcholine binding protein (AChBP) chimera structure 5AFJ. Middle panel, 4F8H: subsite 2 is used by ketamine without a ligand in subsite 1 (which is a proton site in GLIC) in the GLIC structure 4F8H. Right panel, superposition: the color codes match the ligands for each structure in the overlay. Larger ligands like methyllycaconitine can use both sites simultaneously. However, most ligands are observed in subsite 1, as shown in this overlay of six structures that was generated with a secondary structure match superposition. Only fragment 1 in 5AFJ, ketamine in 4F8H and a part of the methyllycaconitine molecule in 2BYR occupy the site 2. Approximately 20 structures with ligands in the ECD interface were screened, and no other instances of site 2 usage were found in this sample (data not shown).

The sites at the ECD-interface have been known long before the first X-ray crystallographic structures revealed their 3D structures (Sieghart, 1995). Many ligands have been assigned successfully to individual ECD-interface subtypes in the past (Hosie et al., 2003; Sieghart, 2015). It has been generally assumed that loops A-G form one binding site, which usually is occupied by one ligand. However, evidence from crystal structures strongly suggests the existence of three subsites at the ECD-interface that can be occupied by small molecules and cations either individually or together. Subsite 1 is formed by amino acid residues from loops B and C of the principal subunit, and from loops E and D from the complementary subunit as seen in the ligand interactions in the 4COF structure (see Figure 4 and “Supplementary Figure 2B”). In all structures that were analyzed here, most ECD-interface ligands are found in subsite 1 (see Figure 4), including the GlyR antagonist strychnine (Du et al., 2015; Huang et al., 2015).

Subsite 2 is formed by parts of loop C and pre-B residues (from strand 7) on the plus side, and by part of the loops G and F on the minus side (see Figure 4). Larger ligands can occupy a space enclosed by loops A, B and C of the principal subunit together with G, D and E, sometimes even F from the complementary subunit, and thus occupy both subsites simultaneously. This is the case for the methyllycaconitine bound AChBP structure (2BYR, Hansen et al., 2005, see Figure 4). Furthermore, subsite 2 at the ECD-interface apparently can be occupied by a small molecule either alone (ketamine bound GLIC) or together with another molecule that occupies subsite 1 (lobeline plus fragment 1 in 5AFJ). Yet another variation on multiple ligands at the ECD-interface has been reported for the AChBP (Stornaiuolo et al., 2013, PDB ID 4BFQ), where multiple copies of the same ligand are bound “behind” loop C (Stornaiuolo et al., 2013). Whether multiple ligands can occupy the ECD-interface at the same time in GABA_A_ receptors cannot be evaluated on the basis of X-ray crystallographic structural data, as benzamidine occupies only subsite 1 with a single molecule. However, the evidence from all templates together with the homology models suggest that both subsites should exist in GABA_A_ receptors and may be used by small ligands independently, or by larger ligands that should occupy the whole volume. Thus, in computational docking studies it might be important to consider which part of the interface is likely to harbor the small molecule of interest, or if it is possible that several subsites are occupied simultaneously. These findings may offer a structural explanation for the observation that additional ligands can bind with unchanged affinity to receptors into which the photoaffinity ligand flunitrazepam has been covalently incorporated (McKernan et al., 1998).

Subsite 3 harbors a Ba^2+^ ion in the ELIC structure (Zimmermann et al., 2012) where it displays inhibitory effects. A homologous site was described earlier based on mutagenesis data in GABA_A_ receptors (Hosie et al., 2003) as a binding site for zinc ions. The binding site was proposed to be located in the α+/β− interface and the binding site forming residues were α1E137, α1H127 (both on β strand 7) and β3E182 (on loop F). The homologous amino acids to β3E182 in the other subunits are uncertain due to the ambiguity of the F loop alignment (see “Supplementary Figure 3A”). Specific interfaces generally can harbor cation binding sites in this localization.

#### Extracellular Intrasubunit Sites 4–6

Three additional binding sites not localized at subunit interfaces were observed in the ECDs of various structures (see Figure 1, Table 1). An intra-subunit site (site 4) was found to be occupied by flurazepam in ELIC (Spurny et al., 2012) with an access path pointing towards the channel vestibule (see “Supplementary Figure 4”). The same site was also found in other ELIC structures, occupied with glycerol (Pan et al., 2012b) and Ba^2+^ (Spurny et al., 2013), as well in GLIC accommodating acetate (Fourati et al., 2015). Different ions also can bind at this position (Sauguet et al., 2014b). Furthermore, the same pocket was occupied by an allosteric modulator in the nAChR α7-AChBP chimera (fragment 4: 4,5-dibromo-N-(3-hydroxypropyl)-1H-pyrrole-2-carboxamide; Spurny et al., 2015). As this site is observed in many crystal structures of three different proteins (ELIC, GLIC, nAChR- α7-AChBP chimera), and flurazepam exerts effects in GABA_A_ receptors that are consistent with the usage of distinct high and low affinity binding sites (Baur et al., 2008), we wondered if such a site 4 may exist in GABA_A_ receptors as well. The crystal structures and sequence alignments of the anion selective eukaryotic homologs (GluCl, GABA_A_ receptors and GlyRs) reveal that a variable piece between β strands 5 and 5’ is longer in these receptors compared to the proteins in which such an intra-subunit binding site was seen (ELIC, GLIC and the nAChR α7-AChBP chimera; “Supplementary Figures 3A, 4”). This insertion completely occludes the space in which ligands are seen in the homologous proteins (see “Supplementary Figure 4”). Thus this putative site was rejected and not investigated further here.

For the remaining ECD intrasubunit sites 5 and 6 the homology models are fully consistent with pockets in these localizations in GABA_A_ receptor subtypes. The novel ECD- intra-subunit binding site 5 was observed to contain bromoform (an analog of the volatile anesthetic chloroform) in ELIC (Spurny et al., 2013). Very recently, the same site was shown to be occupied with xenon in GLIC (Sauguet et al., 2016). Site 5 is located in the packing core between the ECD inner and outer sheets near the disulfide bridge (see Figure 1). Close inspection of this region in GABA_A_ receptor homology models that are based on the GABA_A_ receptor crystal structure 4COF (Miller and Aricescu, 2014) suggests that indeed small hydrophobic molecules could in principle find enough space to bind in this region. The putative pocket forming amino acids are indicated in the master alignment (“Supplementary Figure 3A”). At this time, no evidence exists which allows assignment of any known GABA_A_ receptor ligands to this site.

In the nAChR α7-AChBP chimera yet another allosteric binding site that is formed in part by the N-terminal α helix (helix 1, see site 6 in Figure 1) was observed (Spurny et al., 2015). The existence of site 6 was also confirmed experimentally in the nAChR where its ligand was shown to act as an negative allosteric modulator (Spurny et al., 2015). The amino acid residues putatively forming site 6 in GABA_A_ receptor subunits are highlighted in the master alignment (“Supplementary Figure 3A”) and indicate some potential subunit specificity. Although so far no experimental evidence exists that points to such a site in any GABA_A_ receptor subtype, many ligands act at so far unidentified sites (Sieghart, 2015) and site-directed mutagenesis experiments can be designed on the basis of our models in order to investigate further. This site would have the advantage to be directly located in a single subunit—thus selective ligands would act at a single subunit, rather than at an interface. Their affinity and efficacy thus would not depend on two pocket forming subunits, which would make ligands of site 6 very versatile tool compounds. Ligands acting at this site in the nAChR were shown to exert inhibitory effects (i.e., act as negative allosteric modulators or noncompetitive antagonists, Spurny et al., 2015). For many GABA_A_ receptor subtypes no (allosteric or orthosteric) selective antagonists exist, thus a new class of noncompetitive allosteric antagonists would be most useful.

#### TMD Inter-Subunit and Intra-Subunit Sites Near the Junction with the ECD

The 4COF structure has no ligand bound to the TMD, while several crystal structures feature very diverse ligands bound to different places in this domain (see Figure 1 and Table 1). There is agreement that the GABA_A_ receptors’ TMD-interfaces (site 7) harbor binding sites for ligands (for example etomidate, barbiturates, avermectin), in part with considerable specificity for certain interfaces (Chiara et al., 2012, 2013; Jayakar et al., 2014; Middendorp et al., 2015; Maldifassi et al., 2016). A zinc binding site in the TMD involving β3H267 and β3E270 has also been reported (Hosie et al., 2003).

**Figure 5:**
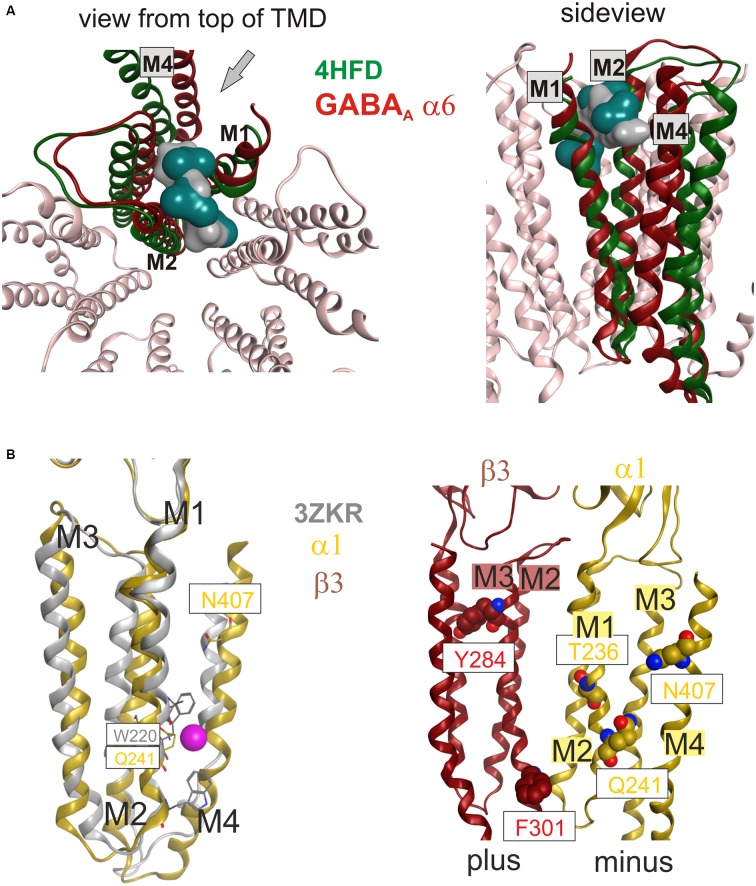
**(A)** Top and side views of the TMD of a GABA_A_ receptor model (α6 subunit red, other subunits light pink) superposed with a single subunit of the bromoform bound GLIC F14’A 4HFD structure (green). The pocket volume in 4HFD is occupied by three bromoform molecules (dark cyan volume) in the GLIC structure at an intra-subunit site mainly formed by M1 and M2. The homologous pocket volume near α6I228 can accommodate a furosemide molecule (gray volume). The side view is rendered from the outside of the channel between M1 and M4. The viewing perspective is indicated in the left image with an arrow. **(B)** Localization of amino acids implicated in neurosteroid action relative to sites 9 and 10. The left panel displays a superposition of a single subunit’s TMD of the bromoform bound ELIC structure 3ZKR (gray) with a homology model of a GABA_A_ receptor α1 subunit based on 4TNW (yellow). Only a single Br atom of the bromoform has been identified in the crystal structure, it is localized in a groove-like pocket between M1 and M4 on the minus side of the TMD and displayed here as a magenta space-filling atom. Among the binding site forming residues in GLIC is W220, the homologous residue in the GABA_A_ receptor α1 subunit is Q241. The right panel depicts amino acids implicated in actions of neurosteroids at or near β+/α− interfaces along the whole length of the TMD (Hosie et al., 2006; Bracamontes et al., 2012; Chen et al., 2012).

However, so far it remained unclear if other binding sites exist in GABA_A_ receptors’ TMD. Some sites have been proposed on the basis of combined mutational and modeling studies (Sigel et al., 2011; Baur et al., 2013), or by integrating data from mutational and photoaffinity studies into computational docking approaches (Alvarez and Estrin, 2015). Here we approach this question based on ligand bound structures of homologous proteins. Sites for bromoform, xenon and other ligands were found in an intra-subunit localization in the TMD of GLIC (Nury et al., 2011; Sauguet et al., 2013, 2016; see Figure 1 and “Supplementary Figure 2”). Such an intra-subunit site (site 8) has also been observed for halothane and etomidate with a combined photolabeling/modeling approach in nAChRs (Forman and Miller, 2011). No ligands have been identified in GABA_A_ receptors for any such site this far. On the other hand, for many ligands the binding sites are unknown, as is the case for the α6− selective negative allosteric modulator furosemide. A selectivity determinant for furosemide is the M1 amino acid α6I228 (Thompson et al., 1999). Mapping of the pockets that exist in GLIC (Nury et al., 2011; Sauguet et al., 2013) onto α6-subunit containing homology models reveals that α6I228 is homologous with an amino acid that participates in binding of bromoform and of desflurane in GLIC (Nury et al., 2011; Sauguet et al., 2013; see Figure 5A). Homology models reveal that such a pocket in an α6 subunit would contain amino acid residues from M1, M2, and M3 (see also “Supplementary Figure 3A”). To clarify if the pocket also exists and is large enough to accommodate ligands in GABA_A_ receptors, we did not rely only on the homology with GLIC, but investigated the site 8 region in several homology models. In α6β3γ2 homology models based on 4COF, as well as on 3JAE, 3JAD and 4TNW an intra-subunit pocket is localized in this space which actually can accommodate the furosemide molecule (see Figure 5A) and the position of the hydrophobic α6I228 is in a localization which is consistent with α6− selective action of ligands using this site (all other subunits have a polar amino acid in the homologous position).

It is worth noting that sites 7 and 8 are formed by overlapping parts of the TMD helices, and amino acid sidechains can be localized near access to both sites. Moreover, both sites are occupied simultaneously in a bromoform bound GLIC structure (see “Supplementary Figure 2” and Table 1). Mutational analysis has pointed at involvement of the region near sites 7 and 8 in the β1 subunit in the action of salicylidene salicylhydrazide (SCS) which has been identified as a β1 selective negative allosteric modulator. Amino acids T255 (T230 in M1 of mature protein) and I308 (I283 in M3 of mature protein) have been shown to be crucial for its β1 subtype selectivity (Thompson et al., 2004). Oddly, they do not seem to be part of a single pocket, but rather are approximately 15 Å apart and nearly on opposite sides of the β1 subunits’ TMD (“Supplementary Figure 5”). Thus, it seems unlikely in the light of the structural evidence that these two selectivity determinants form a pocket together. The M3 position is localized on the plus side of the trans-membrane interface, very close to the etomidate pocket of the homologous β2,3 subunits (Chiara et al., 2012). It has been shown recently that binding sites containing this part of the β+ TMD can confer negative allosteric modulation (Estrada-Mondragon and Lynch, 2015), thus it is conceivable that an SCS binding site indeed is localized at the TMD β1+ interface. On the other hand, given the close proximity of the M1 residue to the region in which we localized the putative furosemide site in the α6 subunit, another possible interpretation would be that an SCS site is in an intra-subunit localization, and the access to this pocket is determined by the M3 residue. Yet another possibility would be that both pockets are occupied and together elicit the functional effects. All these hypotheses can be tested experimentally on the basis of the structural models presented here.

#### Lipid and Putative Steroid Sites in the TMD

In site 9, a localization between M1 and M4 of a single subunit (see Figure 1), a molecule of bromoform was found to bind in ELIC (Spurny et al., 2013). In the GluCl structure 3RHW the homologous position is occupied by a lipid fragment (Hibbs and Gouaux, 2011). We thus investigated whether a ligand binding site could exist in the homologous position of any GABA_A_ receptor subtype. A ligplot analysis of the bromoform bound structure together with homology models of GABA_A_ receptor subtypes containing an α1 subunit revealed that the ELIC residue W220 that is engaged in hydrophobic interactions with bromoform is homologous with α1Q241 (see Figure 5B). This amino acid was identified in mutational studies to be essential for the positive modulatory action of neurosteroids (Hosie et al., 2006). It was later demonstrated that the region around α1Q241 can be transposed to the β or γ subunit and induces steroid sensitivity in chimeric constructs (Bracamontes et al., 2012). An additional amino acid that was implicated in the modulatory action of neurosteroids is residue α1N407 on M4 (Hosie et al., 2006). Previous localizations of these residues based on a cryo- EM structure of the nAChR (Unwin, 2005; Hosie et al., 2006) in homology models were interpreted to form an intrasubunit pocket. In the homology models based on the GABA_A_ receptor structure (Miller and Aricescu, 2014) the amino acids of the α1 subunit (Q241 on M1 and N407 on M4) that govern positive modulatory action of neurosteroids are positioned around site 9 which is a groove at the subunit’s minus side where in the crystal structures bromoform and lipid binding are observed (see Figure 5B), rather than forming an intrasubunit pocket (see “Supplementary Figure 6”). These results so far suggest that neurosteroids may exert part of their complex actions on α1− containing receptors by binding to a TMD-M1/M4 site of the α1 subunit that is in the localization of site 9 in the crystal structures (see Figure 5, Table 1).

In β3 homopentamers, a photoreactive steroid labeled β3F301 (Chen et al., 2012), indicating that while potentiation appears to be α dependent (Bracamontes et al., 2012), steroid binding also occurs in β3 homopentamers. The β3F301 is homologous with β2L301, which was shown to participate in the modulatory effects of anandamide and 2-AG on the GABA_A_ receptors containing the β2 subunit (Sigel et al., 2011; Baur et al., 2013). Mutational analysis and computational docking studies indicated that 2-AG modulates GABA_A_ receptors by binding at a localization which here is called site 10 (Sigel et al., 2011; Baur et al., 2013). The residues contributing to this binding site are β2M294, β2L301 and β2V302 on M3; β2S429, β2F432, β2F433 and β2V436 on M4 (see “Supplementary Figure 3A”), and the homologous position contains a lipid molecule in the GLIC 3EAM structure (Bocquet et al., 2009). These observations suggest that neurosteroids and endocannabinoids can have common or partially overlapping binding sites on GABA_A_ receptors, and that neurosteroids can possibly bind to multiple sites in the TMD—namely sites 9 in alpha subunits and 10 in beta subunits.

The functional role of site 10 cannot be deduced based on this data. However, multiple sites have been proposed previously (Hosie et al., 2006) as different amino acids compared to those that mediate modulatory effects, namely α1T236 (M1) and β3Y284 (M3) seem to be implied in direct activation of GABA_A_ receptors by steroids (Hosie et al., 2006). In previous models based on a cryo-EM structure of the nAChR (Unwin, 2005) these amino acids were proposed to form a common pocket at the TMD β+/α− interface (Hosie et al., 2006), however, in models based on 4COF this is not the case (see Figure 5B and “Supplementary Figure 6”).

It seems that the effects of inhibitory steroids are elicited by a completely different mechanism (Seljeset et al., 2015). Thus, the total number of steroid binding sites on individual GABA_A_ receptor subytpes is unclear at this time. Previously it was suggested on the basis of computational docking that an interface pocket localization between the β+ and the α− surfaces near the ICD would form one of the elusive steroid binding sites (Alvarez and Estrin, 2015). While such a binding site localization is not directly supported by any experimental structure, its possible existence cannot be rejected on the basis of the lack of a structure with a ligand in such a position. However, the structural evidence (Bocquet et al., 2009; Hibbs and Gouaux, 2011; Ulens et al., 2014) together with the data from mutational analysis (Bracamontes et al., 2012) and photoaffinity work (Chen et al., 2012) renders sites 9 and 10 as promising candidate sites for modulatory effects (site 9), and activating effects (site 10), but fails to identify the role of α1T236 in activating effects and provides no clue on the inhibitory effects. Thus further studies will be needed to clarify, and the models presented here can aid in the design of future studies.

#### Summary of Binding Sites Proposed to Exist in GABA_A_ Receptors

**Figure 6:**
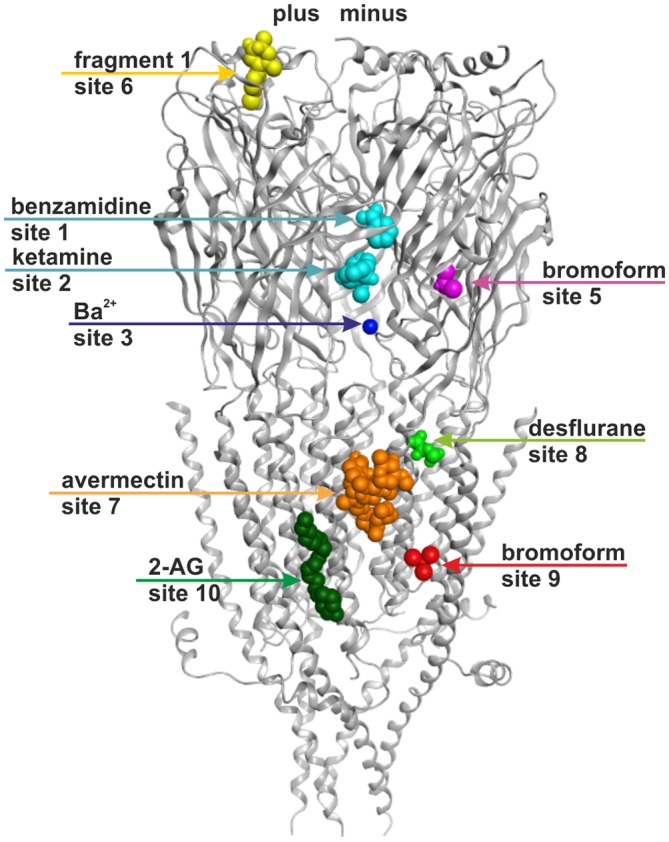
**Binding sites in GABA_A_ receptor homology models based on structural evidence from homologous proteins.** The image shows a homology model based on 4COF (ECD, TMD) and 4PIR (ICD) with one representative ligand per representative site: benzamidine in site 1 and ketamine in site 2 (cyan), Ba^2+^ in site 3 (dark blue), bromoform in site 5 (right subunit, magenta), fragment 1 in site 6 (left subunit, yellow), avermectin in site 7 (orange), desflurane in site 8 (green), bromoform in site 9 (right subunit, red), 2-arachidonglycerol (2-AG) in site 10 (left subunit, dark green). Note that each of the sites could occur in five subunits or at five interfaces, respectively, but may display ligand specificity. Some sites may not exist in all subunits or at all interfaces. Amino acids putatively forming these sites are color coded in the alignment shown in “Supplementary Figure 3A”.

Figure 6 provides an overview of the localizations of the already previously known (1, 3, 7, 10) and the putative new (2, 5, 6, 8, and 9) allosteric sites in a model of a GABA_A_ receptor. The model is based on 4COF in the ECD and TMD, and includes the fragment of the ICD observed in the 4PIR structure, which is modeled according to this structure. The binding sites were mapped to this dual template model to visualize their localizations relative to the three domains and the subunit interfaces. The pocket mapping results (see “Supplementary Figure 3A”) can be utilized to design mutational studies for the investigation of sites 1, 2, 3, 5, 6, 7, 8, 9 and 10 in any subtype of the GABA_A_ receptor.

### Impact of Conformational Changes on the Structure of Binding Sites

In a next crucial step we investigated the impact of conformational changes on the structures of the binding sites. To study any binding site structure in more detail and to perform computational docking or virtual screening experiments using homology models, it is necessary to assess which of the available structures are the most suitable templates, and to clarify if several templates have to be considered in order to study the consequences of protein motion on a site of interest. Flexible regions or regions that undergo strong motions or adapt ligand induced fit states may not be in the desired conformation in the 4COF structure which likely represents an agonist bound desensitized state (Miller and Aricescu, 2014).

#### Conformational Changes in the GlyR and the GluCl

In order to determine how the structure of individual sites changes during conformational transitions between states, we generated homology models from two series of structures that represent diverse ligand bound and conformational states. Cryo- EM strutures of the GlyR α1 subtype have been obtained bound with glycine (an agonist, 3JAE), strychnine (an antagonist, 3JAD) and a combination of glycine and avermectin (3JAF; Du et al., 2015), and a crystal structure of the GlyR α3 subtype in complex with strychnine (5CFB; Huang et al., 2015) is also available. Together, these enable researchers to study conformational changes and induced fit from agonist (glycine), antagonist (strychnine) and TMD-interface ligand (avermectin) binding. The second series that was utilized here to study the impact of protein motion on the different pockets of interest comprises the GluCl structures which additionally include an apo state (Hibbs and Gouaux, 2011; Althoff et al., 2014), see Table 1.

#### Domains, Subunits and Pentamers Compared within and across GluCl, Glycine Receptors and the GABA_A_ Receptor Structures: Local and Global Motion, Conserved and Variable Structure Elements

**Figure 7:**
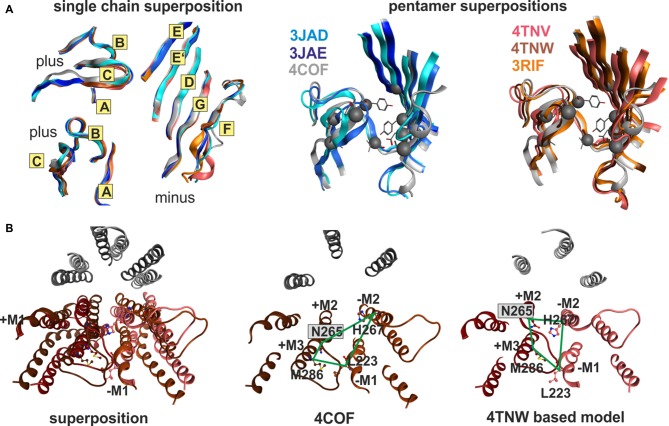
**Impact of protein motion on the ECD and TMD interface pockets. (A)** ECD binding site forming segments in models based on the indicated six structures. The left panel shows the very close structural overlap that results from a superposition of the ECDs of single chains. The plus side forming segments A, B and C are shown from two different perspectives, the minus side is depicted once. The color codes match the PDB IDs. The right panel shows the distinctly interface geometries in the pentameric structures. The 4COF based interface (gray) is compared with models based on agonist (3JAE) and antagonist (3JAD) bound GlyR template structures (blue/ cyan/ gray), as well as with apo- (4TNV) and differently ligated GluCl structures (red/orange/brown). Every interface has a unique geometry due to the near rigid-body like movements which the ECD and TMD perform relative to one another during conformational changes, see also “Supplementary Figures 7, 8”.** (B)** The TMD interface in two different protein conformations contains different pocket forming residues. The left panel shows a superposition of 4COF with a β3 homopentamer model based on 4TNW, only the TMD is displayed. Two complete TMDs are displayed in different hues of red-brown, the plus side subunit is darker. Of the other three subunits, only M2 is displayed in gray for orientation relative to the pore. The four amino acids that were used to quantify differences of interface geometry (see “Supplementary Figure 9”) are shown in stick rendering. The other two panels display 4COF and the 4TNW based model viewed individually from the same perspective as in the superposition, but the far end of the TMD is clipped for more clarity. In the 4COF structure β3H267 is localized in a remote position to the subunit interface. A similar position is also observed in models based on the GluCl structure 3RIF. The 4TNW based β+/β− interface features β3H267 on the minus side of the interface vis a vis from β3N265, consistent with both residues being part of a single TMD-interface pocket. The position of β3H267 relative to the interface is strikingly different in the two protein conformations.

Superposition of individual domains was analyzed to understand how individual domains change during conformational transitions, and to assess similarity between the three analyzed families (GluCl, GlyR, GABA_A_ receptor). The apo (4TNV), the POPC bound (4TNW) and the avermectin bound states (3RIF, 3RIA, 3RHW) possess nearly identical ECD structures, and no “capping” or “uncapping” motion of loop C of the subunit can be detected at all (see Figure 7A and “Supplementary Figure 7B”, see also the analysis of the interfaces below). While the loop C tips of all GluCl structures and of the GABA_A_ receptor β3-subunit overlap very closely (to less than 1 Å), the situation is slightly different for the GlyR structures. Specifically, the ECDs of the strychnine bound GlyR structures 3JAD and 5CFB overlay less tightly with the glycine bound and glycine/avermectin bound stuctures. These two structures do display a small loop C uncapping by approximately 2 Å compared to the glycine bound GlyR structure 3JAE (see “Supplementary Figure 7B”). Additionally, the two strychnine bound structures display complex rearrangements of the entire ECD, including a shift in the position of the N-terminal helix 1 (see “Supplementary Figure 7B”).

The very high degree of overall conformational rigidity of the ECD across the investigated structures reflects in a total root mean square deviation (RMSD) among the α-carbon atoms of 1 Å for the GluCl structures, and 1–1.2 Å for the GlyRs. The high structural conservation among these three families reflects in an ECD RMSD of only 1.5 Å for the ECD superposition of 4COF (representing the GABA_A_ receptor) compared with 3JAD, 3JAE, 3JAF (three of the GlyR structures) and with 3RIF, 4TNV and 4TNW (representative, structurally diverse GluCl structures, see “Supplementary Figure 7C”).

If the TMDs are superposed, an even smaller overall RMSD of 1.2 Å results (see “Supplementary Figure 7C”). Thus, the individual domains are conformationally remarkably rigid, and highly conserved across the analyzed anion channels. This observation justifies the use of the GlyR and to some degree also the GluCl as alternative templates for modeling GABA_A_ receptor subunits to take advantage of a larger choice of ligand bound states and conformations that exist for these proteins.

If the entire subunits are superposed, the overall RMSD increases to 2.1 Å, reflecting mainly the motions of the two domains with respect to each other (“Supplementary Figure 7B”). The ECD and TMD tilt and rotate/twist in relation to each other (Althoff et al., 2014) during conformational changes. These pronounced motions of the ECD and the TMD relative to one another become evident (see “Supplementary Figure 7B”) if domain superpositions are compared with subunit superpositions. For the entire GluCl and GlyR series, the two domains move nearly rigid-body like and the domains themselves show almost unchanged structures in the different states (see Figure 7A and “Supplementary Figure 7”) as discussed above, with only the junction region at the domain interface being slightly distorted in the course of a twisting and tilting motion of the two domains relative to one another.

When the whole pentamer is viewed, the changes in the individual subunit (different tilt and twist angles of the ECD and TMD) add up with changes of subunit position in the pentamer (rotational and tilting motions of the entire subunit with respect to the longitudinal pore axis). Grouped by structural similarity, the structures fall into two groups comprising a mixture of the proteins, that feature similar conformations within the group. Pentamer superposition results in lower RMSD values between the GlyR structures 3JAE and 3JAF, the GluCl structure 3RIF (as well as 3RHW, 3RIA and 3RI5), and the GABA_A_ receptor structure 4COF. These superpose within the range of 1.6–2.0 Å pairwise RMSD, see tabulated values in “Supplementary Figure 7A”. If any of the former are compared with the GlyR structures 3JAD and 5CFB, or the GluCl structures 4TNV and 4TNW, the RMSD is >3Å throughout (see “Supplementary Figure 7A”). Thus, the first group contains GlyR and GluCl structures of similar conformations. The GlyR structures 3JAE and 3JAF are similar to the GluCl structures 3RIF, 3RIA and 3RHW. The GlyR structures 3JAD and 5CFB are, on the other hand, more similar to the GluCl structures 4TNV and 4TNW than to the GlyR structures 3JAE or 3JAF. The benzamidinde bound GABA_A_ receptor structure 4COF is in a conformation most similar, but not identical with 3JAF of the GlyR series and also quite similar to the GluCl structure 3RIF. Thus, the conformational differences seen between 3JAE and the other GlyR structures, as well as between 3RIF and the other GluCl structures, can be extrapolated to GABA_A_ receptor subunits, where similar changes also potentially occur.

#### Binding Sites at Subunit Interfaces in Different Conformations

Next we examined how the differences in pentamer and dimer geometry impact on the binding sites localized at ECD and TMD interfaces. Both the tilt angles and the rotational position with respect to the longitudinal plane through the interface of pocket forming surfaces in neighboring subunits are different in all templates we considered for this analysis (see Figure 7, “Supplementary Figures 7–9”). The twist and tilt of the ECD relative to the TMD translates into a marked change of the angle between the complementary subunits’ minus side and the principal subunits’ plus side that leads to an apparent change of loop C position relative to the minus side (see “Supplementary Figure 7”). It is important to note though that this is a consequence of a nearly rigid-body motion of the entire ECD, and not an isolated motion of loop C. In fact, a seemingly more “uncapped” loop C can be as close to loop D as a seemingly “capped” one (see “Supplementary Figures 7,8”, especially the distances between the tip of loop C and loop D in 4COF and 4TNV).

To quantify differences in relative positioning of all binding site forming segments, distances between α-carbons of pocket forming amino acids were examined (see “Supplementary Figures 8, 9”). For the ECD-interface we evaluated 10 distances between the plus and minus subunits to obtain insight into the different pocket geometries for 4COF, and for models derived from all GlyR structures and from all GluCl structures, respectively. Figure 7 depicts the various ECD-interface geometries that result from using 4COF, 3RIF, 4TNV, 4TNW, 3JAD and 3JAE as templates. From the distances tabulated in “Supplementary Figure 8” it can be appreciated that each ECD-interface has a unique geometry. Interestingly, the strychnine bound structures feature a narrow subsite 2 (as determined by distances between loop A and loops G and F, see “Supplementary Figure 8”) together with a wide subsite 1 (as indicated by distances from loops B and C to loops D and E, see “Supplementary Figure 8”). In contrast, subsite 1 is much narrower in structures that harbor small ligands in this site (see “Supplementary Figure 8”). Models based on the apo-GluCl structure 4TNV display a large separation between loop B and E, but a small distance between the tip of loop C and the minus side (see “Supplementary Figure 8”). From this we conclude that multiple templates should be considered carefully for computational docking studies—and not be chosen based on highest sequence identity. If antagonist bound states are of interest, 3JAD and 5CFB will be the templates of choice. Otherwise, for very small ligands the templates with the smallest pockets 3JAE or 3JAF, or the intermediate 4COF will be appropriate choices, while for larger ligands 4TNW and 4TNV might yield better results.

In addition to the impact of protein motion, structural variability (i.e., structural differences among the different proteins) is also evident. Of the ECD binding site forming regions, strand 9 on the plus (principal) side, the loop F region on the minus (complementary) side, and the region near site 6 display structural variability (see alignment shown in “Supplementary Figure 3A, 7”). While the loop F region does contain a small structurally conserved motif (see Figure 2), the overall structure is quite different among the different proteins (Nys et al., 2013). Structural prediction of loop F and thus of subsites 2 and 3 at the ECD-interface, as well as of site 6, will contain considerable intrinsic uncertainty. To account for this, neither template analysis nor homology modeling alone are sufficient and more sophisticate computational methods are needed.

The selective use of distinct TMD-interfaces by various ligands (Wingrove et al., 1994; Chiara et al., 2012; Luger et al., 2015) also leads to intense interest in structural studies and computational docking for these sites. While so far only the β+/α−, α+/β− and γ+/β− interfaces have been assigned to specific ligands, homologous interfaces with putative pockets exist at each subunit interface. ρ receptors have been reported to be inhibited/negatively modulated by a number of ligands (Belelli et al., 1999; Thomet et al., 2000) that are allosteric positive modulators in αβ, αβγ or αβδ receptors (Mihic et al., 1997; Bormann, 2000; Kaur et al., 2009). The putative trans-membrane interface pockets that may be present in δ-containing receptors have not yet been explored at all, the same holds for θ, ε or π-containing receptors for which the respective subunit arrangements are unknown.

It has been observed and discussed before that protein conformation in the TMD is very crucial for pocket properties, and thus must be considered carefully for docking studies (Franks, 2015). Based on the detailed analysis of across-interface and within-subunit distances (see “Supplementary Figure 9”) of models based on all GlyR structures and all GluCl structures, as well as on the GABA_A_ receptor and the 5HT_3_-R structure, we identified vastly different pocket shapes (see Figure 7B and “Supplementary Figure 9”). The differences between the interface geometries displayed by the individual members of the GlyR series and the GluCl series is much more pronounced in TMD-interface site 7 than in the ECD. Interestingly, the avermectin bound state and the POPC bound state of GluCl display very different TMD-interface geometries (see “Supplementary Figure 9”). However, the avermectin bound GlyR structures overlap very well with the avermectin bound GluCl. Thus, the analysis of different structures demonstrates that the binding sites localized in the TMD-interface (site 7) undergo marked conformational changes.

These conformational changes are so large that the pocket forming amino acids differ among different states. Amino acid β3H267 of the GABA_A_ receptor β3 subunit is site of photoincorporation of *ortho-*propofol diazirine (Yip et al., 2013). It has been argued previously that there might be multiple, conformation-dependent pockets associated with β3H267 (Franks, 2015) to which ligands might bind in a use-dependent fashion. This obviously raises the question which template structure, and thus which pocket conformation to select for homology modeling and subsequent computational docking studies to probe ligand-bound structures. Notably, in homology models based on the 4TNW structure, the β3H267 side chain is part of the minus site of site 7, and in close proximity to the β3N265 on the plus side of the interface. In these models, both these residues clearly are part of a continuous pocket at the interface (see Figure 7B). Previously, based on a more limited collection of structures, β3H267 was assumed to not be part of the site 7 (barbiturate pocket) at the α+/β− or β+/β− TMD-interface (Yip et al., 2013). Since propofol was reported to competitively inhibit photoincorporation of 3H-azietomidate (Chiara et al., 2012) and 3H-R-mTFD-MPAB (Chiara et al., 2013), it ought to bind at an overlapping site. Our analysis of motion at the trans-membrane interface now reconciles these observations by showing that in certain conformations the photolabeled residue β3H267 is part of the pocket containing the β− interface part (barbiturate-pocket), see Figure 7B. This is in agreement with a recent mutational study where the same conclusion was reached (Stern and Forman, 2016). Thus, for computational docking studies multiple templates should be used to sample these different structures, unless it is clear which crystal structure corresponds to the ligand bound state of interest. Use of the avermectin bound 3RIF structure to localize the binding site of valerenic acid in the GABA_A_ receptor α1β2γ2 subtype was reported recently (Luger et al., 2015). In this study the pocket in the 4COF structure, although more homologous, was found too small and a model based on the avermectin bound structure 3RIF was able to accommodate the ligand.

#### Intrasubunit Binding Sites in Different Conformations

In contrast, the pockets that are localized within a single subunit do not display large structural changes due to the fact the subunits’ ECD and TMD behave nearly rigid-body like. Indeed, we find distances between pocket forming α-carbons to vary minimally (less than 1 Å) in pockets 5, 9 and 10. The situation is different for pocket 6 which is formed in part by the conserved helix 1, and in part by loops that differ in structure between the GlyR and the GluCl. Thus, due to the higher similarity between GABA_A_ receptors and GlyRs, the GluCl may not be a valid template to model pocket 6. The region around pocket 6 also responds to antagonist binding, as evidenced by different helix 1 conformations in the strychnine bound GlyR structures, see “Supplementary Figure 7C”. The single subunits also display motion induced changes in the ECD-TMD junction where pocket 8 is localized. While in models based on the structures 4COF (GABA_A_ receptor), 4TNW (GluCl POPC bound state) and 3JAD (GlyR, strychnine antagonist bound with a closed conformation of the TMD) we find α6I228 of the putative furosemide pocket to be orientated consistently towards the intra-subunit space (see “Supplementary Figure 7C”), the shape and size of pocket 8 differs as indicated by changes in α-carbon distances of up to 2 Å. Careful template comparison and the use of multiple templates is thus advisable for structural studies of all ligand binding sites in cys-loop receptors.

## Discussion

Since so far only a single GABA_A_ receptor subtypes’ crystal structure is available, all insight about structural differences between the subtypes, and all information on different protein conformations rests on sequence data and homology models. Here we took advantage of a large number of structures from homologous ligand-gated ion channels to identify putatively conserved structural motifs that should be present in all GABA_A_ receptor subtypes, to gain insight into protein motion, and to examine the influence of all these factors on known and putative ligand binding sites. While the structures we employed vary in the experimental method by which they were determined and in the nominal resolution, the structural properties that were of interest for this work depend largely on backbone conformation. Therefore, structures with lower resolution can be compared directly with structures of higher resolution for the present analysis. This would be different for computational docking or virtual screening studies, for which resolution must be sufficient to provide reliable sidechain coordinates in the binding site of interest.

The high structural similarity between the GABA_A_ receptor structures’ TMD (Miller and Aricescu, 2014) with the remotely homologous 5-HT_3_ receptor (Hassaine et al., 2014) demonstrates impressively that the structural conservation in the cys-loop family is extraordinarily high. The enormous diversity which furnishes each family member and every receptor subtype with unique pharmacological and electrophysiological properties thus stems from a few highly variable domains, such as the extracellular loops C and F on the one hand, and from subtle differences in structural details on the other hand. The influence of the large and variable intracellular domain on the pharmacological properties of receptor subtypes is largely unclear at this time.

The high degree of overall structural conservation allowed the use of multi-template modeling to integrate information from several related proteins into GABA_A_ receptor models. We identified 10 distinct binding pockets for small molecules and cations per subunit to exist in various atomic structures. Four pockets (1, 2, 3 and 7) are located at interfaces, and are thus formed by two subunits together. The remaining six pockets are formed mostly or completely by a single subunit. Of these 10 small molecule binding pockets, four have been already described before to exist in diverse GABA_A_ receptor subunits and at interfaces formed by specific neighboring subunits. Specifically, sites at the extracellular interface (subsite 1, or subsites 1 and 2 together and the cation binding site 3) and at the TMD-interface near the ECD have already been assigned to various ligands (Sieghart, 2015). In addition, a site formed by parts of the M3 and M4 helices close to the ICD of the β2 subunit has been described as interaction site for the endocannabinoid signal molecules such as anandamide and 2-AG (Sigel et al., 2011; Baur et al., 2013).

By constructing homology models on multiple structures, and by mapping binding sites from more remote homologs (such as GLIC and ELIC) onto the homology models based on 4COF, the GlyR and the GluCl, we investigated also the novel sites 4, 5, 6 and 9. Of the sites that so far have not been described in GABA_A_ receptors, we determined that site 4 (localized at the channel vestibule) which was observed in three homologous proteins (Spurny et al., 2012, 2015; Fourati et al., 2015) does not exist in GABA_A_ or GlyRs. Site 5, a small hydrophobic space within the packing core of the ECD near the disulfide bridge may accommodate small hydrophobic molecules, but we were unable to assign candidate ligands to this site as no experimental evidence such as mutational studies point at the putative pocket forming residues as mediating effects of any ligand. Of interest is the putative existence of site 6, a cavity formed by the N-terminal helix 1 and loop regions which has been described for the nAChR α7−AChBP chimera and a nAChR. Ligands that bind to this site in the nAChR act as negative modulators (Spurny et al., 2015). In GABA_A_ receptor subunits, the pocket forming amino acids would differ strongly between certain subunits, and thus this putative pocket would be suitable for subtype selective targeting of selected subunits. Furthermore, ligands acting at this site would be selective for a single subunit, as opposed to ligands that act at an interface-site which imparts selectivity towards both pocket forming subunits. While no ligand assignment was possible for any of the newly proposed pockets in the ECD, for the TMD-intra-subunit site 8 we identified furosemide as a strong candidate ligand for the α6 subunit’s site 8. Site 9 which is formed by M1 and M4, likely together with the lipid bilayer, is a candidate for the modulatory site of neurosteroids. This site is very similar to the endocannabinoid site 10 (Baur et al., 2013).

We found that protein motion has a large impact on the pockets contained at subunit interfaces, but much smaller effects on intra-subunit sites. Thus, templates which feature different conformational states of the ECD such as the strychnine bound glycine receptor structures (Du et al., 2015; Huang et al., 2015) need to be considered if larger ligands or ligands that exert antagonistic effects shall be docked into the homolgy models. However, regions that are subject both to sequence variability and conformational motion, such as loops C and F, or the junction zone that connects the ECD and TMD, cannot be modeled with high accuracy on any of the available structures at this time and may in addition to sampling the templates broadly require post-modeling procedures such as loop sampling (Sali and Blundell, 1993) and experimental data guided model ranking (Richter et al., 2012; Middendorp et al., 2014) prior to performing advanced computational docking studies or virtual screens.

Analysis of the same region of interest, such as site 7, in different experimental structures has led to important insights regarding the interpretation of sidechain positions with respect to “pockets” or other protein surfaces. Not only limited accuracy of low resolution structures, but also pronounced difference in conformational states can result in controversial sidechain localization. This was exemplified for residues implicated in steroid action (Hosie et al., 2009) as well as in binding sites used by anesthetic compounds (Yip et al., 2013; Franks, 2015; Stern and Forman, 2016). Studies in which structural data is utilized should therefore never be restricted to “the closest homolog”, or “the highest resolution structure”, instead, the available breadth of information needs to be integrated to arrive at reasonable estimates of possible uncertainty.

We also presented the first models that incorporate a large ICD fragment. Overall, the models presented here help to localize amino acid residues that have been identified as crucial for ligand effects by biochemical means (Thompson et al., 1999, 2004; Hosie et al., 2006; Carland et al., 2009; Chen et al., 2012; Baur et al., 2013; Estrada-Mondragon and Lynch, 2015) and to explore their structural vicinity. This leads to testable hypotheses regarding the role of specific amino acids in ligand effects. In the interpretation of the models and the putative pocket contributing segments (see “Supplementary Figure 3A”) the limitations inherent to the method need to be kept in mind. The strictly conserved parts of the backbone that can be deduced from appropriate 3D superpositions will be the most reliable parts of structural models, while more variable regions and INDELS will be of limited reliability. Whether a difference in amino acids between subtypes or species can reliably be utilized for computational work such as docking depends on its localization on conserved or variable parts. Thus, comparing different structures thoroughly and in detail is invaluable also to deal with the limits in model accuracy.

## Author Contributions

ME supervised the study design. CS, RVF, RP, MH and ME analyzed atomic structures. RP, XS, ME, MH and RVF generated and analyzed GABA_A_ receptor models. ME and GFE supervised computational work. The manuscript was written by RP, MH and ME.

## Funding

Financial support by Austrian Science Fund through the graduate school program Molecular Drug Targets MolTag (FWF, Grant No. W1232) to RP and the FWF project P27746 to MH and XS is acknowledged.

## Conflict of Interest Statement

The authors declare that the research was conducted in the absence of any commercial or financial relationships that could be construed as a potential conflict of interest.

## Footnotes

^1^http://www.rcsb.org/pdb/

^2^http://www.ebi.ac.uk/msd-srv/ssm/

^3^http://www.uniprot.org/

^4^http://www.clustal.org/

^5^http://salilab.org/modeller/

^6^http://www.ebi.ac.uk/msd-srv/ssm/
