# Supplementary material for: Structural Studies of GABAA Receptor Binding Sites: Which Experimental Structure Tells us What?
Source: Front Mol Neurosci. 2016 Jun 16;9:44. doi: 10.3389/fnmol.2016.00044 (PMC4910578; doi:10.3389/fnmol.2016.00044)
Supplement: Supplementary file 1 [file Image_1.pdf]

Supplementary Material

Structural studies of GABA$_A$ receptor binding sites: Which crystal structure tells us what?

Roshan Puthenkalam, Marcel Hieckel, Xenia Simeone, Chonticha Suwattanasophon, Roman V. Feldbauer, Gerhard F. Ecker and Margot Ernst*

*** Correspondence:** Margot Ernst: margot.ernst@meduniwien.ac.at

Supplementary Figures

1: Schematic representation of pentameric arrangement in αβγ, αβ and αβδ receptors

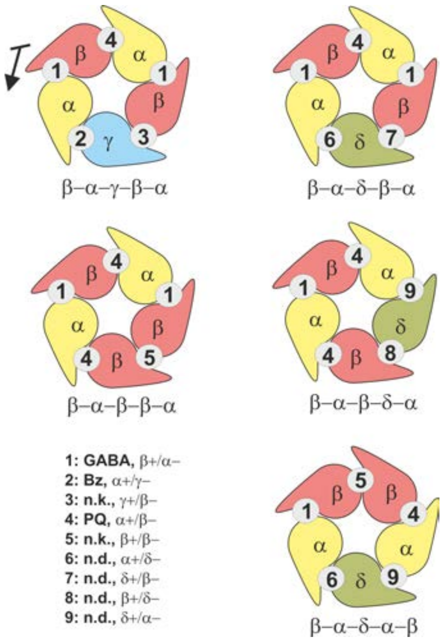

Supplementary Figure 1. Subunit arrangements and naming convention for αβγ, αβ and αβδ receptors. Receptor schemes are drawn such that a GABA binding site is always the top left "site 1". The arrangement is written starting with the top left subunit as indicated by the arrow in the αβγ receptor. The tip of each subunit represents the plus side/ loop C as seen from extracellular. All interfaces that occur in confirmed or proposed receptors are listed in the legend. GABA is the ligand for the β+/α– interface; Bz indicates the high affinity benzodiazepine site; pyrazoloquinolinones (PQ) have been shown to allosterically modulate GABA$_A$ receptors by binding to α+/β– interfaces. No known (n.k.) endogenous or drug-like ligands exists for the γ+/β– and β+/β– interface (although benzamidine was found to bind to the β+/β– interface and have agonistic effects there (Miller and Aricescu, 2014)**)**, but the existence of these interface is considered confirmed. For the δ subunit, the existence of δ+/β–, δ+/α– as well as α+/δ– and β+/δ– has been investigated (Barrera *et al.*, 2008; Kaur *et al.*, 2009; Sigel *et al.*, 2009; Eaton *et al.*, 2014), but none have been demonstrated to exist in native receptors so far (n.d.). Here three arrangements that have been discussed for these receptors are depicted. (Barrera *et al.*, 2008; Kaur *et al.*, 2009; Sigel *et al.*, 2009; Eaton *et al.*, 2014)**.**

2: Small ligand binding sites in crystal structures

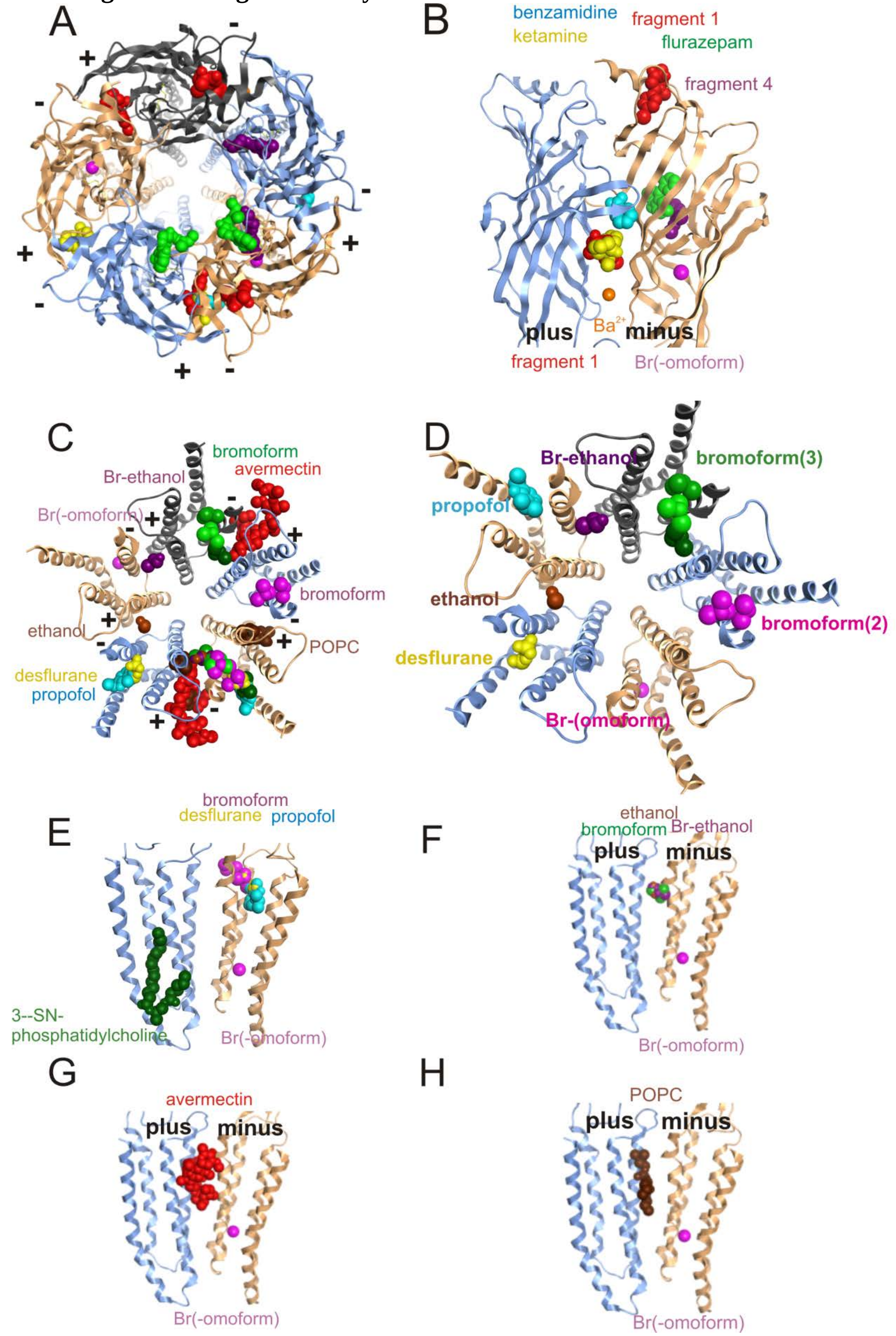

See next page for legend

Supplementary Figure 2a. Small ligand binding sites found in X-ray structures of pentameric cys-loop receptors. The figure shows a superposition of selected structures (with the PDB IDs shown in boldface) from Table1, see manuscript Table 1. All panels show the subunits of 4COF in ribbon representation, the subunits are depicted in alternating colors for a better visualization of the subunit interfaces. The ligands are depicted in space-filling representation. **A:** Top view of all superposed structures with ligands in the ECD. **C, D:** Top view of structures with ligands in the TMD (ECD removed). **C** and **E** show that the propofol binding site is different from the site used by desflurane or bromoform. Side view of two subunits' ECD (**B)** and TMD **(E, F, G** and **H)** with different ligands. Side views of the TMD showing single ligands and groups of ligands that occupy either intra-subunit sites (**E**) or different positions at the interface (**F, G** and **H**) together with the bromoform seen in a position between M1 and M4 in ELIC (magenta) and 3-SN-phosphatidylcholine between M3 and M4 in GLIC (green) (only shown in **E**). In the ELIC structure 3ZKR, only the Br atom of the bromoform was resolved both in the ECD site (**B**) and the M1/M4 site (**E-H**). Bromoform molecules which were found in a mutated GLIC at the interface (green, **C, D** and **F**) and in the wild type in an intra-subunit site (magenta, **C, D** and **F**) are highlighted in two different colors to be better distinguishable. In **F** only one of three bromoform molecules is displayed to show the overlap with the (Br-)ethanol site.

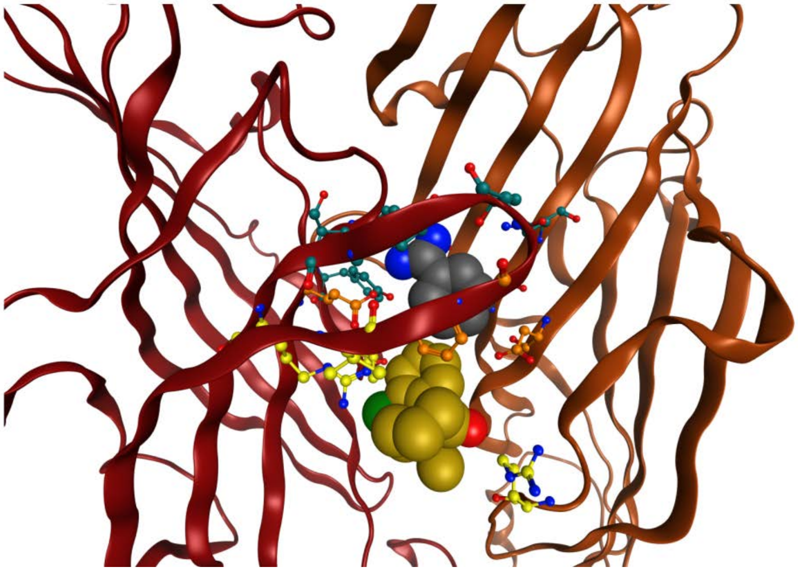

Supplementary Figure 2b. The extracellular interface can harbor ligands in two distinct subsites. Amino acids exclusive to subsite 1 are depicted in cyan, residues involved in forming both sites in orange, and residues exclusive to subsite 2 in yellow. All subsite assignments are based on 4COF and a mapping of the 4F8H in which ketamine is bound to subsite 2 onto a β3 model.

3: Methods workflow and alignments used for homology modeling and pocket mapping

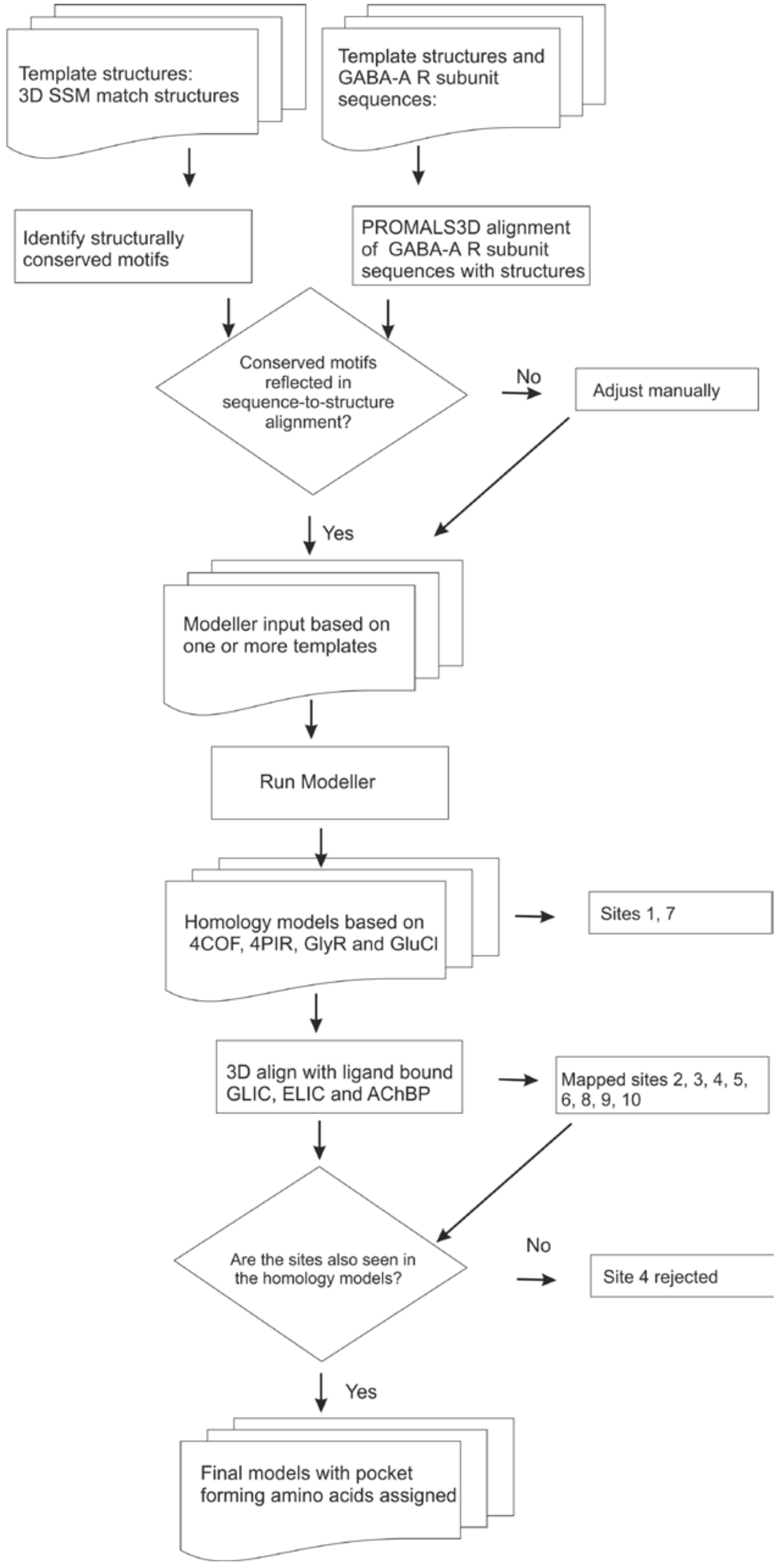

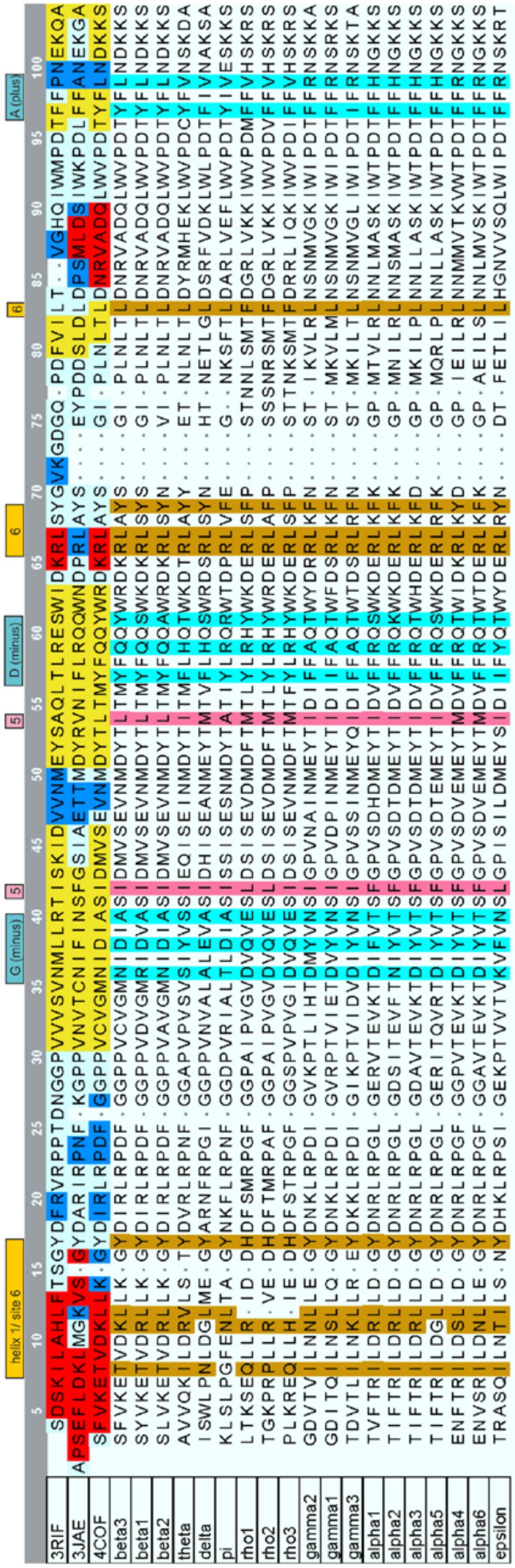
helix 1/ site 6
G (minus)
5
D (minus)
6
A (plus)
3RIF
3JAE
4COF
beta3
beta1
beta2
theta
delta
pi
rho1
rho2
rho3
gamma2
gamma1
gamma3
alpha1
alpha2
alpha3
alpha5
alpha4
alpha6
epsilon

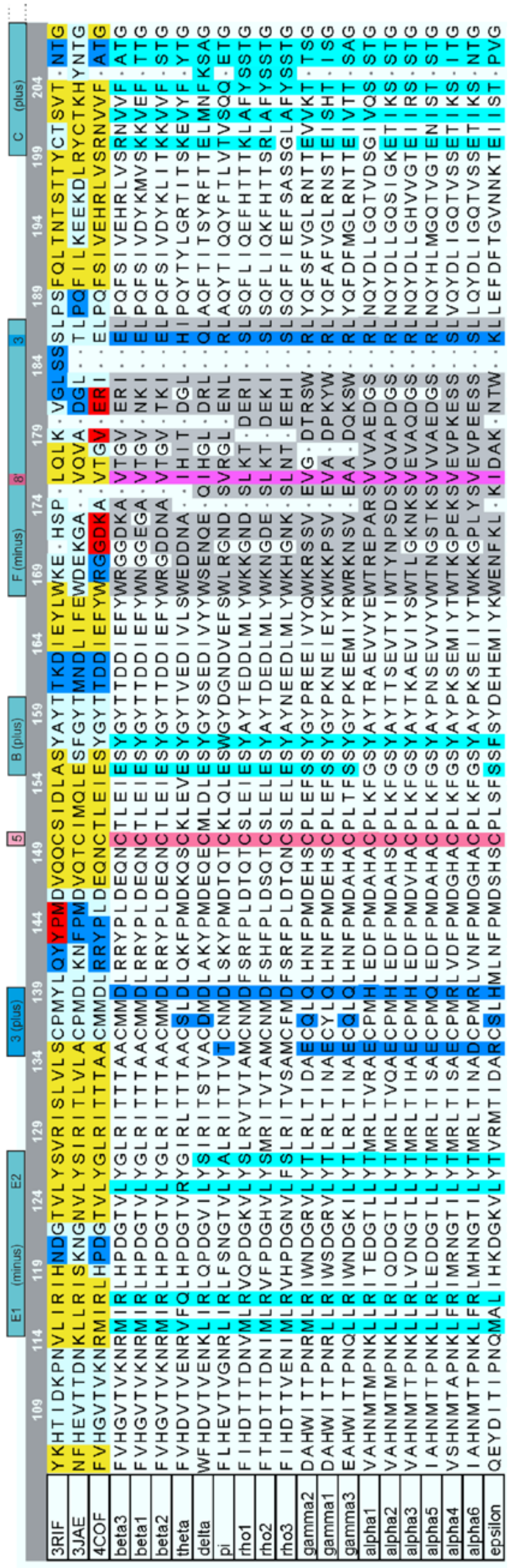
E1 (minus)
E2
3 (plus)
5
B (plus)
F (minus)
8'
3
C (plus)
3RIF
3JAE
4COF
beta3
beta1
beta2
theta
delta
pi
rho1
rho2
rho3
gamma2
gamma1
gamma3
alpha1
alpha2
alpha3
alpha5
alpha4
alpha6
epsilon

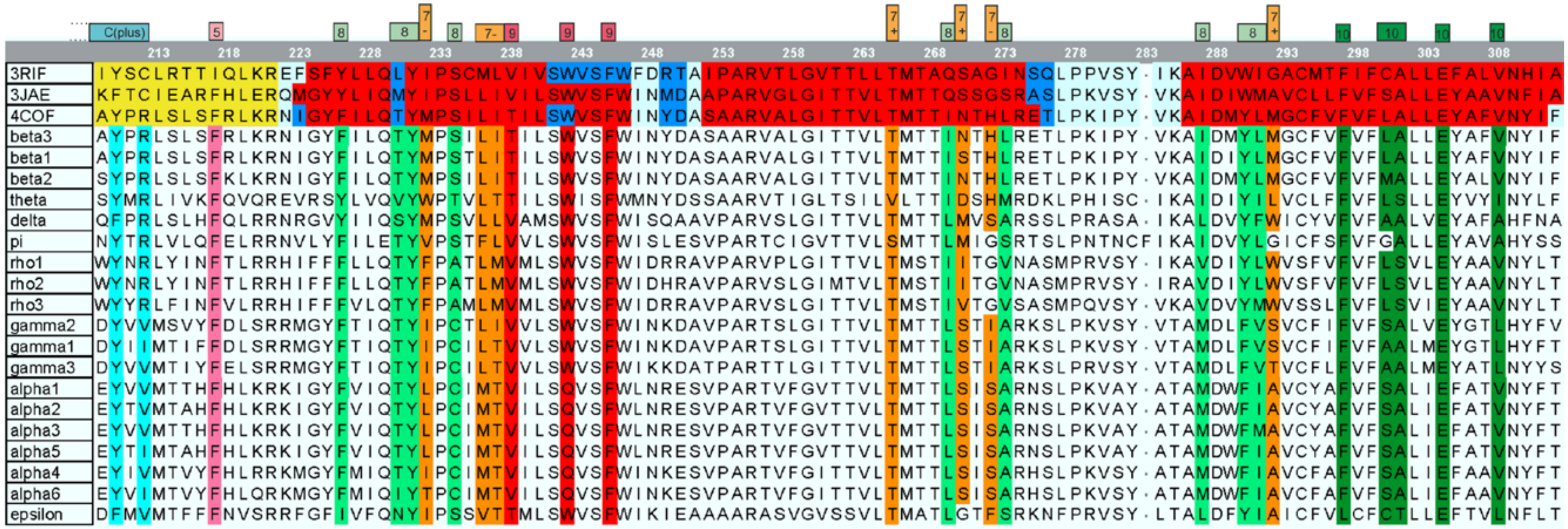

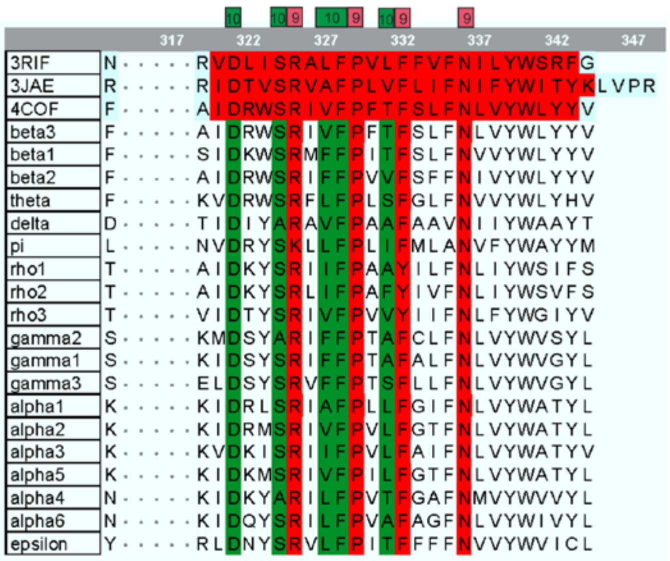

Supplementary Figure 3a.

Master alignment resulting from PDBeFold superposition of three crystal structures (top three sequences, 3RIF, 3JAE and 4COF) and Promals3D alignment of the nineteen $GABA_A$ receptor subunits (truncated N-terminally and between M3 and M4) as it was used to generate homology models based on 4COF, and on GlyR and GluCl crystal structures respectively. The rat sequences were used except for theta, where only the mouse had the Swiss-prot reviewed label. The color codes in the top three sequences denotes secondary structure (yellow: beta strand; red: helical; blue: turn), while in the remaining sequences the binding site forming amino acids are highlighted by color coding matched to Figures 1 and 6 in the manuscript. A legend for the binding site forming segments is given above the sequence alignment. All plus and minus sides of sites 1 and 2 are indicated in light blue except loop F residues are highlighted in gray to indicate low structural equivalence. The alignment is optimized to correctly model strand 8', as indicated by the magenta marked position in loop F. For a specific site, the binding site forming amino acids can differ from these shown here due to structural and conformational variability, the assignments made here are based on the analyzed template structures and, where possible, on mutational evidence together.

Color legend for Supplementary Figure 3a:

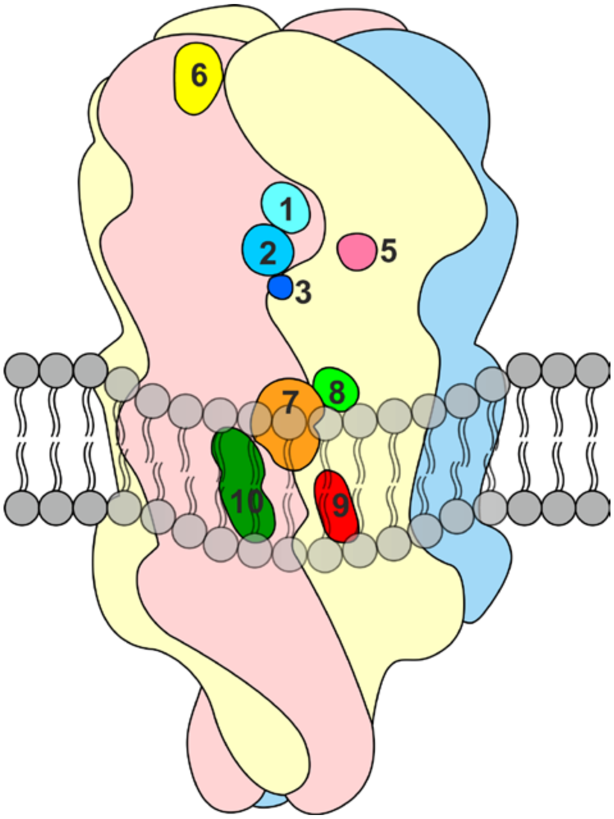

Schematic rendering of a heteropentameric $GABA_A$ receptor (space filling proportions) with "prototypical" binding sites, each site shown only in one subunit/ interface. These sites potentially can occur in each subunit (at each interface, some may be limited to specific subunits or interfaces). For example, the cation site 3 is not present in all combinations of plus (principal) and minus (complementary) sides. Binding site numbering corresponds to the main text. In the alignment (Supplementary Figure 3a) the amino acids which likely contribute to these sites are color coded in matching colors, with some notable exceptions:

Sites 1 and 2: These subsites of the ECD- interface are lined by amino acids from segments ("loops") A, B, C, D, E, F and G. A, B, C, D, E and G are color coded in blue in the alignment, no distinction is made between subsites 1 and 2. Segment (loop) F is coded in gray due to intrinsic variability in loop F, which doesn't allow to assign individual amino acids as "pocket forming" on the basis of homology models.

Site 3: Not all subunits have sidechains in the homologous positions to the known cation site that are suitable for cation chelation, thus, the dark blue coding is only present on some subunits.

Sites 5, 6, 7, 8, 9 and 10: The color codes rely on pocket mapping, and thus the list of amino acids that contribute to a specific pocket in a given subtype may be longer or shorter. Techniques such as pocket finding and site exploration should be applied to study individual situations and must augment the information given in Supplementary Figure 3a.

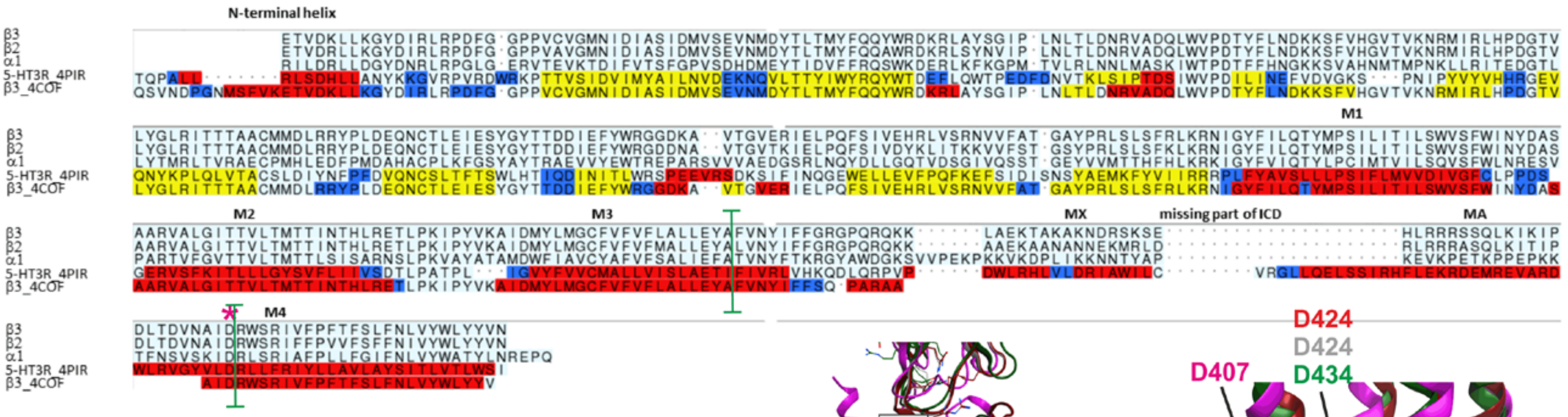

Structural match of TMD and ICD between 4PIR, 4COF and 2BG9

Chains	1	2	3	4
1:2bg9_a.A		3.64	3.71	3.66
2:4cof_a.A	3.64		2.67	0.65
3:4pir_a.A	3.71	2.67		2.62
4:model_b3.A	3.66	0.65	2.62	

Total C-alpha RMSD for superposition of M1, M2 and M3 of the four depicted structures

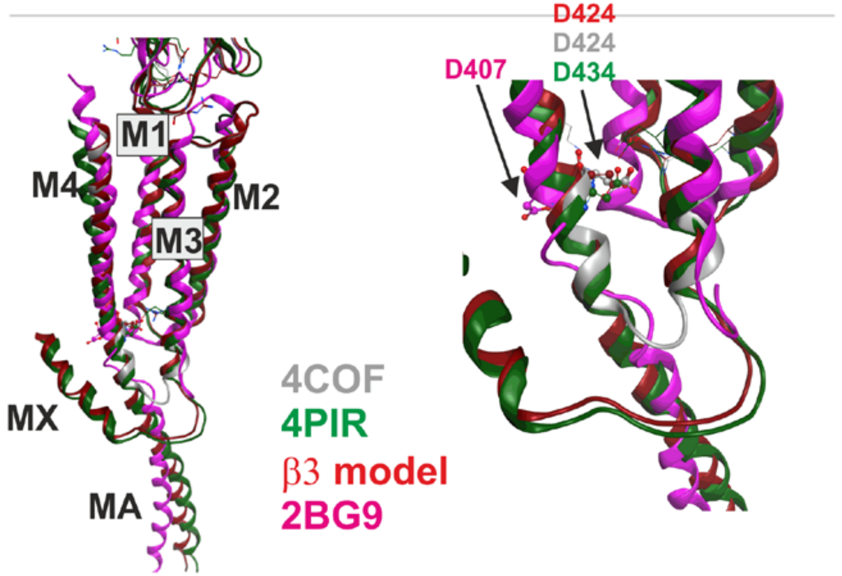

Supplementary Figure 3b. PROMALS3D alignment of the sequences of the α1, β2 and β3 GABA$_A$R subunits with the structure of 5-HT$_3$R (PDB code: 4PIR). The missing part of the ICD in 4PIR is indicated above the alignment. For the GABA$_A$R subunits only a part of the MA helix is predicted, thus, in the alignment only the part of the ICD sequence is shown for which a sequence-to-structure prediction is available. The linker between M3 and MX is predicted to be of variable length. The colors in the sequences of 4PIR and 4COF refer to the secondary structures elements: α helices (red), β strands (yellow) and turns (blue). A magenta asterisk indicates the conserved acidic position in M4 in the alignment. Green bars indicate the borders between 4COF and 4PIR template usage.

Models: A superposition (using M1, M2 and M3 to superpose) of chain A of 4PIR (green), 4COF (gray), a GABA$_A$ R β3 model based on 4COF and 4PIR as indicated in the alignment (see methods, red), and chain A of 2BG9 (magenta) is shown. The RMSD values are indicated in the table. The conserved M4 Asp (D) amino acid that is indicated in the alignment with a magenta asterisk is shown in stick rendering in the enlarged view. Notably, while the sidechains in 4PIR and 4COF overlay well, the D407 of the 2BG9 structure is off.

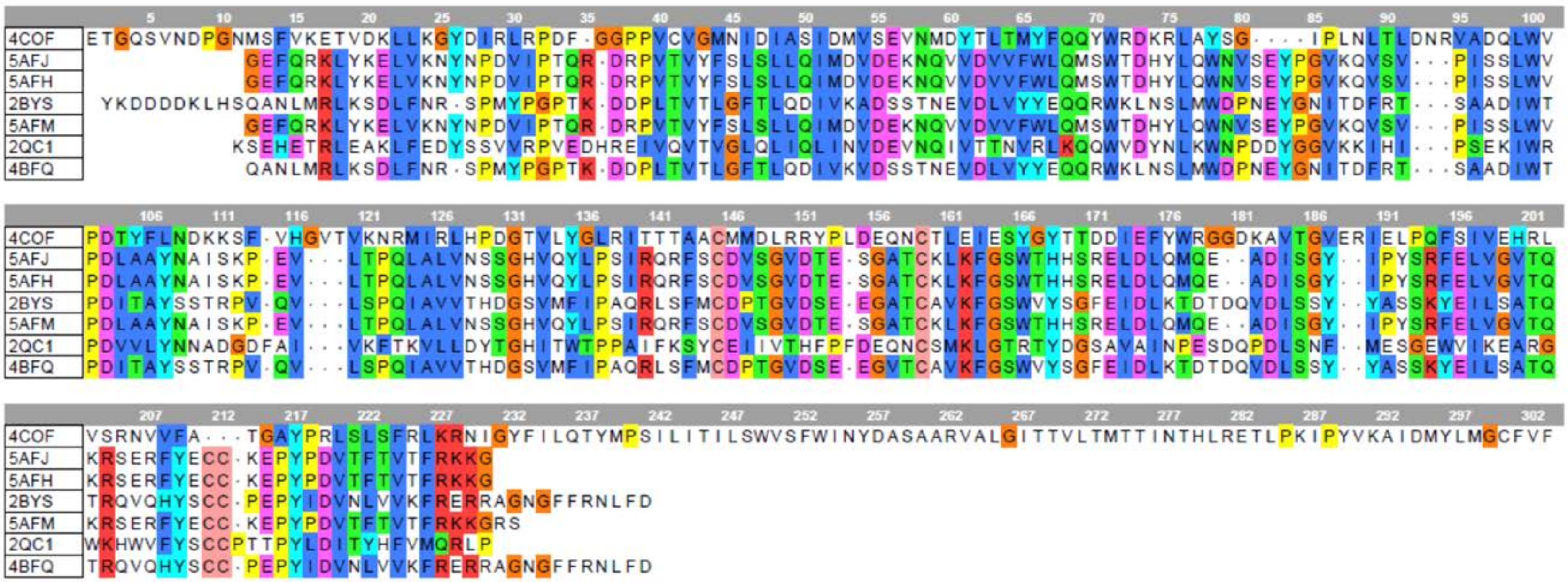

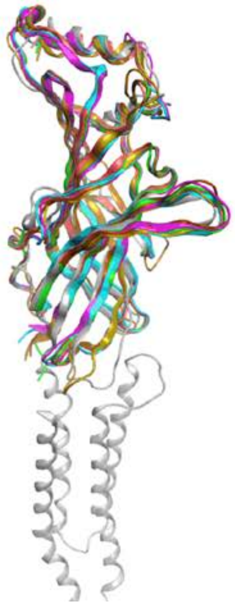

Supplementary Figure 3c. PDBeFold alignment of 4COF with AChBP, nAChR and nACh-R-AChBP chimeras. Only incomplete TMD of 4COF is shown. The alignment was used for the mapping of pocket 6 onto homology models of $GABA_A$ receptors. The structural view shows 2QC1 (gold), 5AFJ (red), 5AFH (brown), 2BYS (cyan), 5AFM (green) and 4BFQ (magenta). All structures superpose to 2.1 Å, which corresponds to an ECD superposition because all AChBP and nAChR structures lack the TMD. The RMSD between the 4COF ECD and the shown structures ranges from 2.5 Å to 3.0 Å.

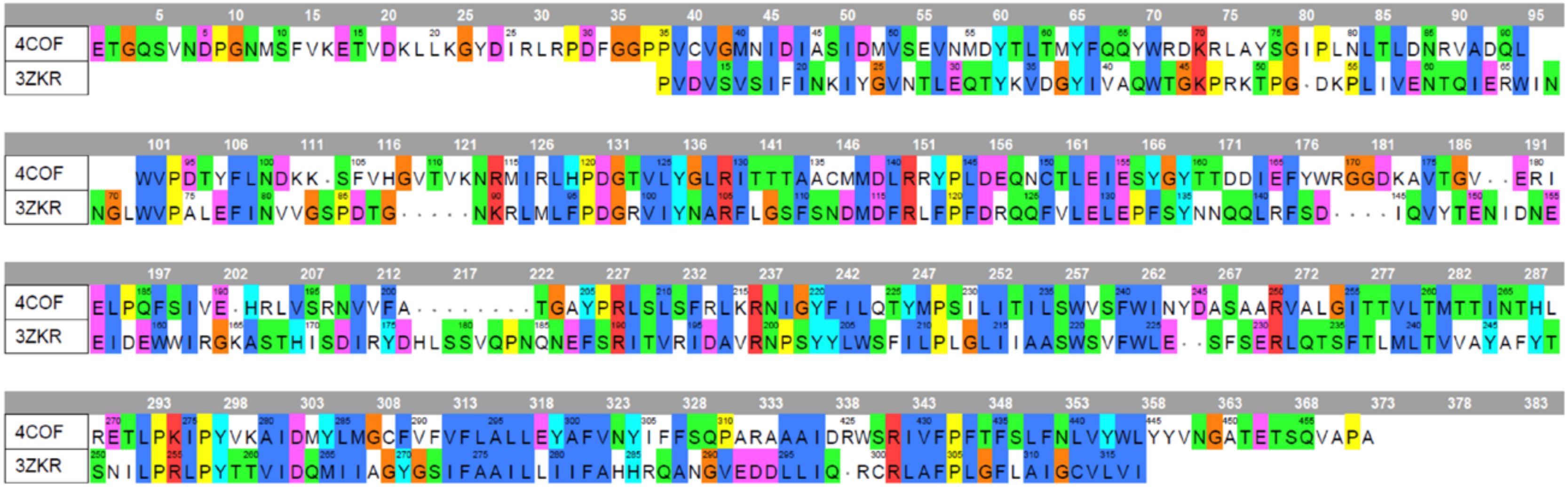

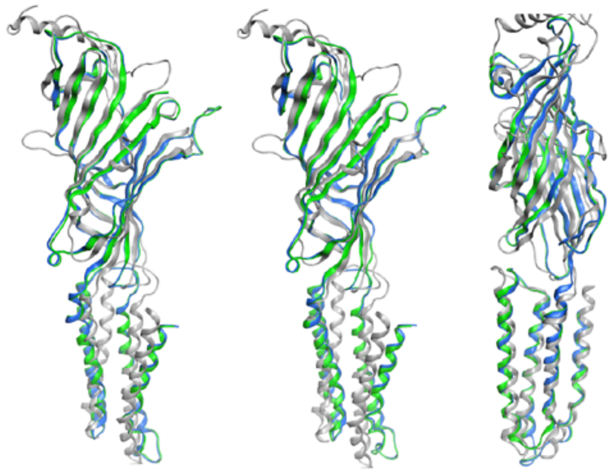

Supplementary Figure 3d. PDBeFold alignment of 4COF (gray ribbon) with ELIC structures 2YOE (blue ribbon) and 3ZKR (green ribbon). The alignment was used for the mapping of pockets 5 and 9 onto homology models of $GABA_A$ receptors. Overall RMSD values of subunit fit, ECD fit and TMD fit are provided below the ribbon images that illustrate these three fits. Pairwise RMSD 4COF- 2YOE/ 3ZKR is 2.7 Å in the ECD, and 3.0 Å in the TMD. Thus, the pocket mappings for pockets 5 and 9 contain some uncertainty, and the pocket 5 and 9 markings in Supplementary Figure 3a may feature /or lack amino acids that are/ are not pocket contributing. Pocket topology is likely to be conserved and also confirmed by the very recent xenon bound GLIC structure (Sauguet et al. 2016).

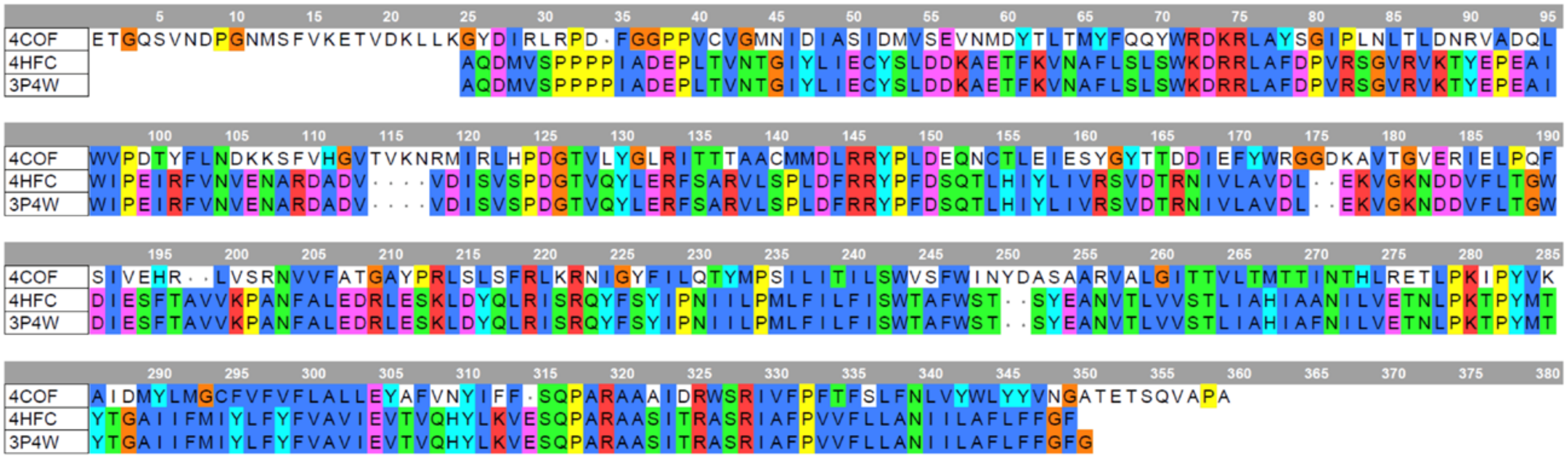

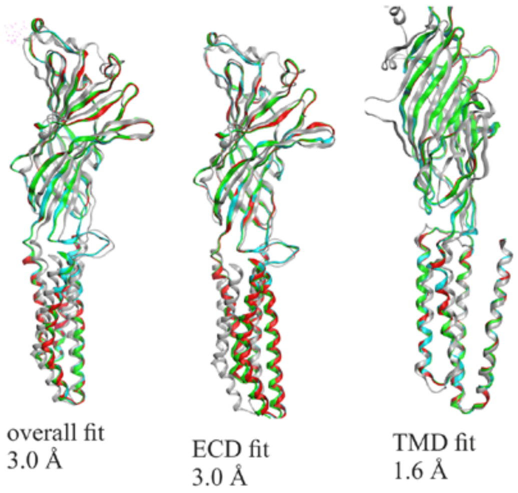

Supplementary Figure 3e. PDBeFold alignment of 4COF (gray ribbon) with GLIC structures 4F8H (green ribbon), 4HFE (red ribbon) and 3P4W (cyan ribbon). The alignment was used for the mapping of pockets 2 and 8 onto homology models of $GABA_A$ receptors. Pairwise RMSD 4COF- GLIC is 3.0 Å overall for all GLIC structures, 3.0 Å in the ECD, and 1.6 Å in the TMD. Due to the high similarity of the TMD between GLIC structures and 4COF, the mapping of pocket 8 would be expected to be accurate.

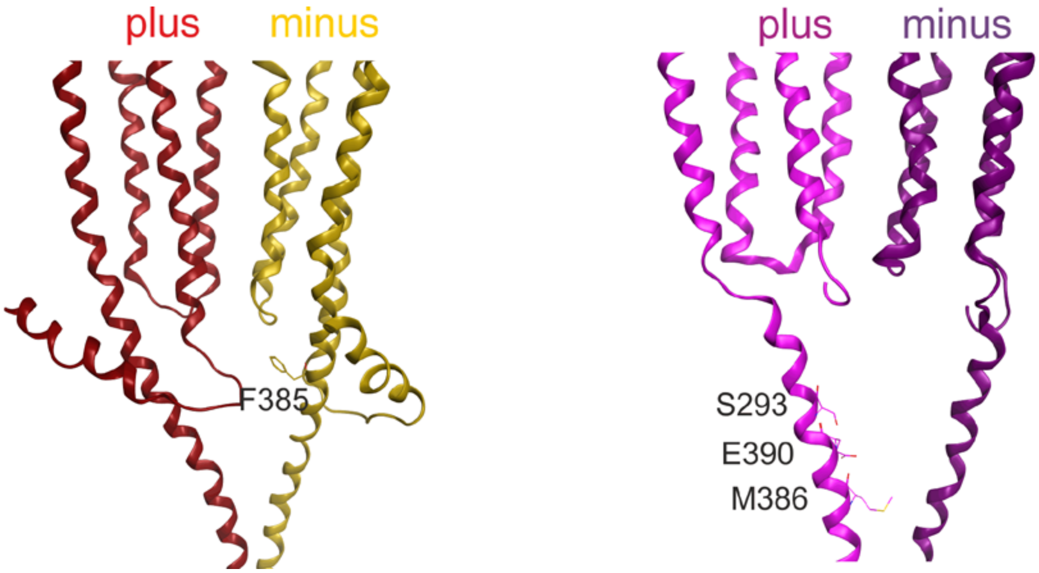

Supplementary Figure 3f: The position of the α1F385 on the minus face of the MA helix in the 4PIR based ICD model of the $GABA_A$ receptor as shown in Figure 3 in the MS, in comparison with the amino acids in 2BG9 on the plus face of the MA helix that have been reported to be labeled by photoreactive ligands (Chiara et al. 2009a, b).

Description of Supplementary Model: Alignment used for the Modeller input:

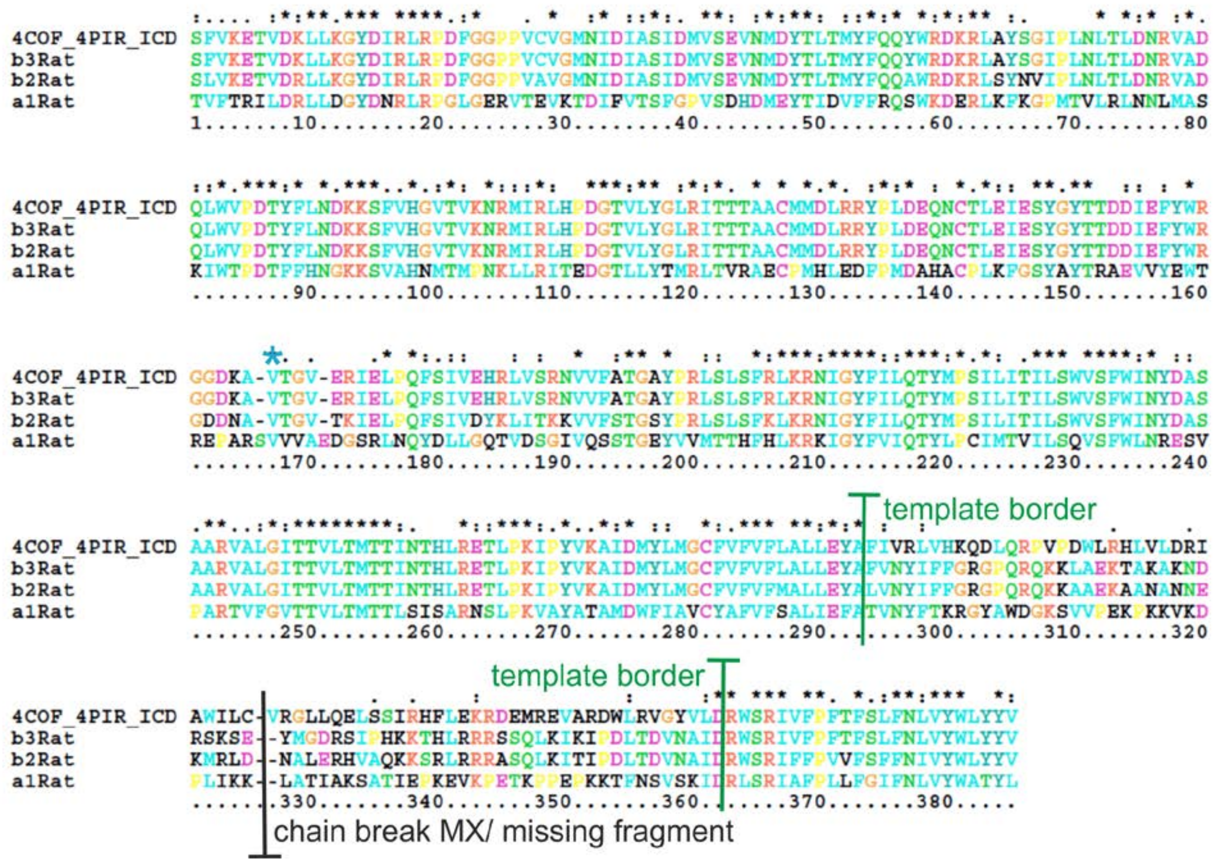

This alignment variant was used to generate the model depicted in Figure 5 of the main manuscript and supplied as supplementary model, note that all sequences are trimmed.

Note that a "virtual chimera" 4COF-4PIR-ICD is shown to indicate the ECD and TMD fragments based on 4COF, and showing 4PIR sequence only between the green bars that denote the template borders. The model is an α1β3 subtype with the following chain composition:

Chain A: β3 subunit fragments
Chain B: β3 subunit fragments
Chain C: α1 subunit fragments
Chain D: β3 subunit fragments
Chain E: α1 subunit fragments
The loop F insertion for the α1 subunit was built as indicated by the alignment in the region 165 – 175, the blue asterisk * denotes the conserved start of strand 8`, see also Supplementary Figure 3a. Note that the model is a raw homology model, loop refinement must be done for the insertions prior to further processing (energy minimizing, debumping etc.) and using the model. β2 sequence is included for easy reference for site 10 defining residues (2-AG site).

4: Mapping of site 4 from ELIC and AChBP onto GABA$_A$ receptor models rejects this site

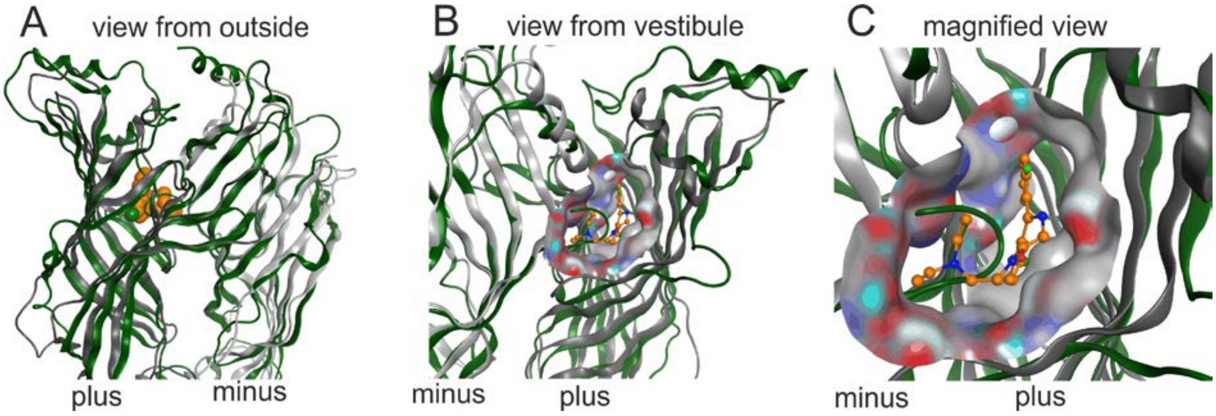

Supplementary Figure 4. The ELIC (2YOE) structure is shown in gray, and the GABA$_A$R β3 structure in green. Panel **A** shows a side view from the outside of the channel. In panel **B** and **C** the view is from inside the channel mouth into the intra-subunit pocket to see the flurazepam molecule in the subunit on the right (which is the plus-subunit's backside). **B:** The left (minus) subunit makes also some contacts with flurazepam. The GABA$_A$R structure can be seen to have a small so-called bulge (pre E loop insertion) at the interface between the two subunits which clashes with the position of the flurazepam in this aligned view. This bulge is conserved among GABA$_A$ receptor subunits, and is also present in the GluCl and GlyR. **C:** The bulge occupies the space which is filled with flurazepam in the ELIC.

5: Amino acids implicated in the β1 selective effects of salicylidene salicylhydrazide

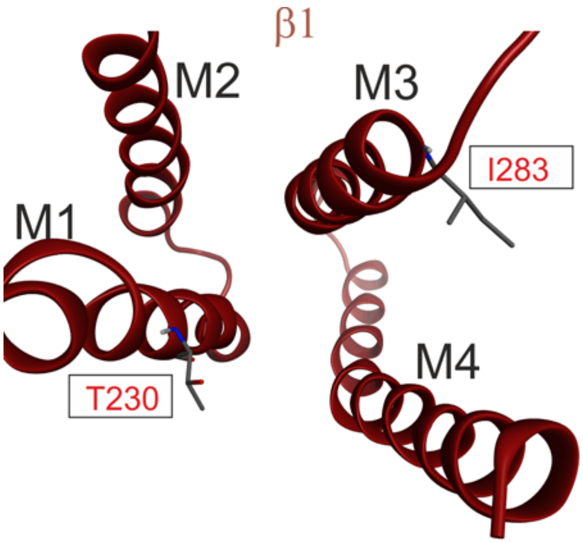

Supplementary Figure 5. Amino acids implicated in the β1 selective effects of salicylidene salicylhydrazide (SCS) in a view from the top of the subunits' TMD. These amino acids are unique to the β1 subunit (see Supplementary Figure 3a), and both impact on SCS action upon mutational analysis.

6: Amino acids implicated in the modulatory and direct actions of neurosteroids

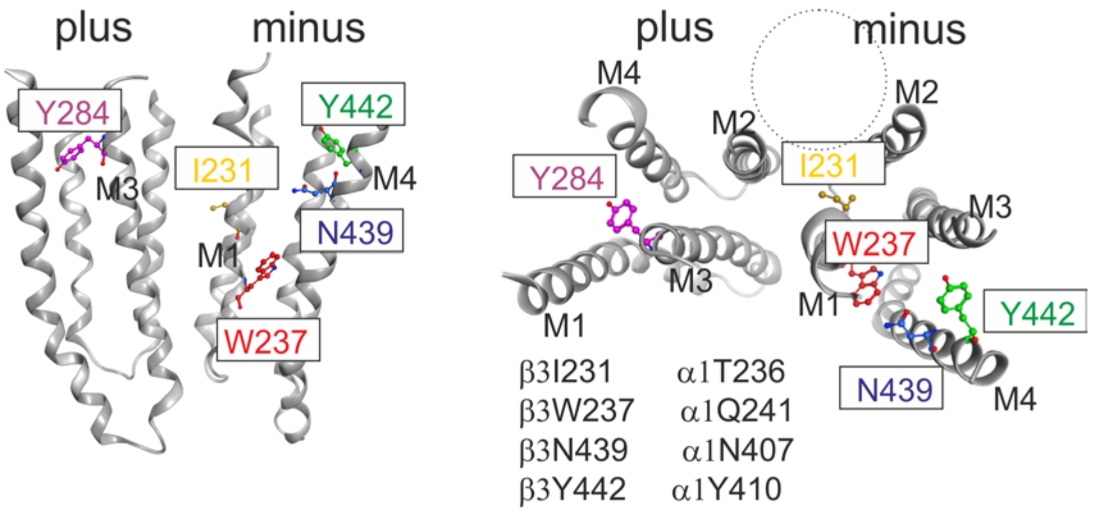

Supplementary Figure 6. Amino acids discussed in Hosie et al. 2006 as forming two distinct binding sites: Two TMDs of the 4COF structure (β3 homopentamer) are visualized as ribbons, seen from "outside the pore" in the left panel, and from the top of the TMD in the right panel. The indicated amino acids are rendered in colors similar as in the Supplementary Figure 4 in Hosie et al. 2006. A lookup table for the corresponding α1 homologues is also provided.

Note the following different localizations in this structure compared to the model based on 2BG9 from Hosie et al. 2006: (All amino acid numbers refer to 4COF, see above lookup table for the corresponding α1 positions)

- M3 Y284 is not localized at the subunit interface
- M1 I231 (α1 T236) is localized in an intra-subunit position, not at the subunit interface
- The three amino acids on the minus side have been proposed to form an intra- subunit pocket based on the 2BG9- derived model. The 4COF structure suggests rather a position lining a lipid exposed groove on the surface of the M1/M4 boundary, consistent with a binding site formed partly by M1 and M4, and partly by lipid constituents (site 9). However, it cannot be excluded that steroid binding will induce an intrasubunit pocket which so far has not been observed in any experimental structure. The domain rigidity of the TMD (see Supplementary Figures 7a- 7c) however can be interpreted as evidence against the existence of a conformation with a significantly cavity in this localization.

7: Protein motion/ conformational changes

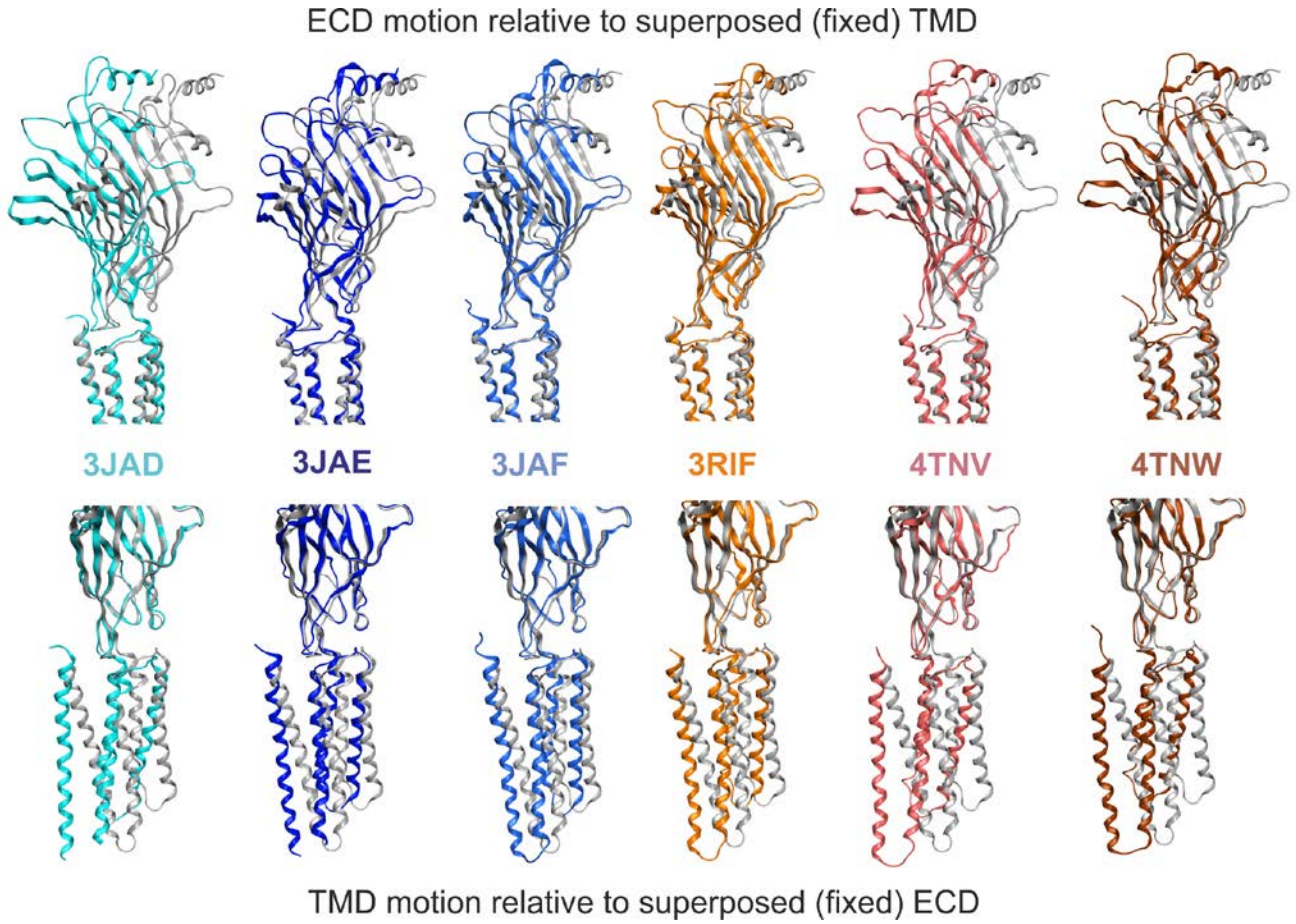

Alpha C RMSD for whole pentamer superposition (global fit)						
	**3JAF**	**3JAE**	**3JAD**	**3RIF**	**4TNW**	**4TNV**
**4COF**	1.7	2.0	3.0	2.0	3.7	4.2
**3JAF**		1.0	2.9	1.5	3.2	3.6
**3JAE**			3.0	1.9	3.1	3.8
**3JAD**				2.8	2.4	2.2
**3RIF**					2.6	2.9
**4TNW**						1.9

Supplementary Figure 7a: **The structures** show a pairwise comparison between 4COF subunits (gray) and representative GlyR and GluCl structures. The top row shows the subunits with superposed TMDs, illustrating the different tilt and twist angles that the ECDs resume with respect to the TMDs. The bottom row shows the subunits with superposed ECDs, illustrating the different tilt and twist angles the TMDs have with respect to the ECDs. Notably, each of these structures has a unique overall conformation, thus, each subunit possesses five or more distinct conformational states. The avermectin bound GlyR (3JAF) and the avermectin bound GluCl (3RIF) display very similar structures. **The table** indicates the overall Cα RMSD differences from a global pentamer superposition (the superpositions per domain yield much lower RMSD values, see main text) as an indication for conformational similarity. Blue cells are GlyR- GlyR comparisons, light brown cells GluCl- GluCl comparisons, light purple cells GABA$_A$R- GlyR comparisons, green cells GABA$_A$R- GluCl comparisons, and yellow cells GlyR- GluCl comparisons.

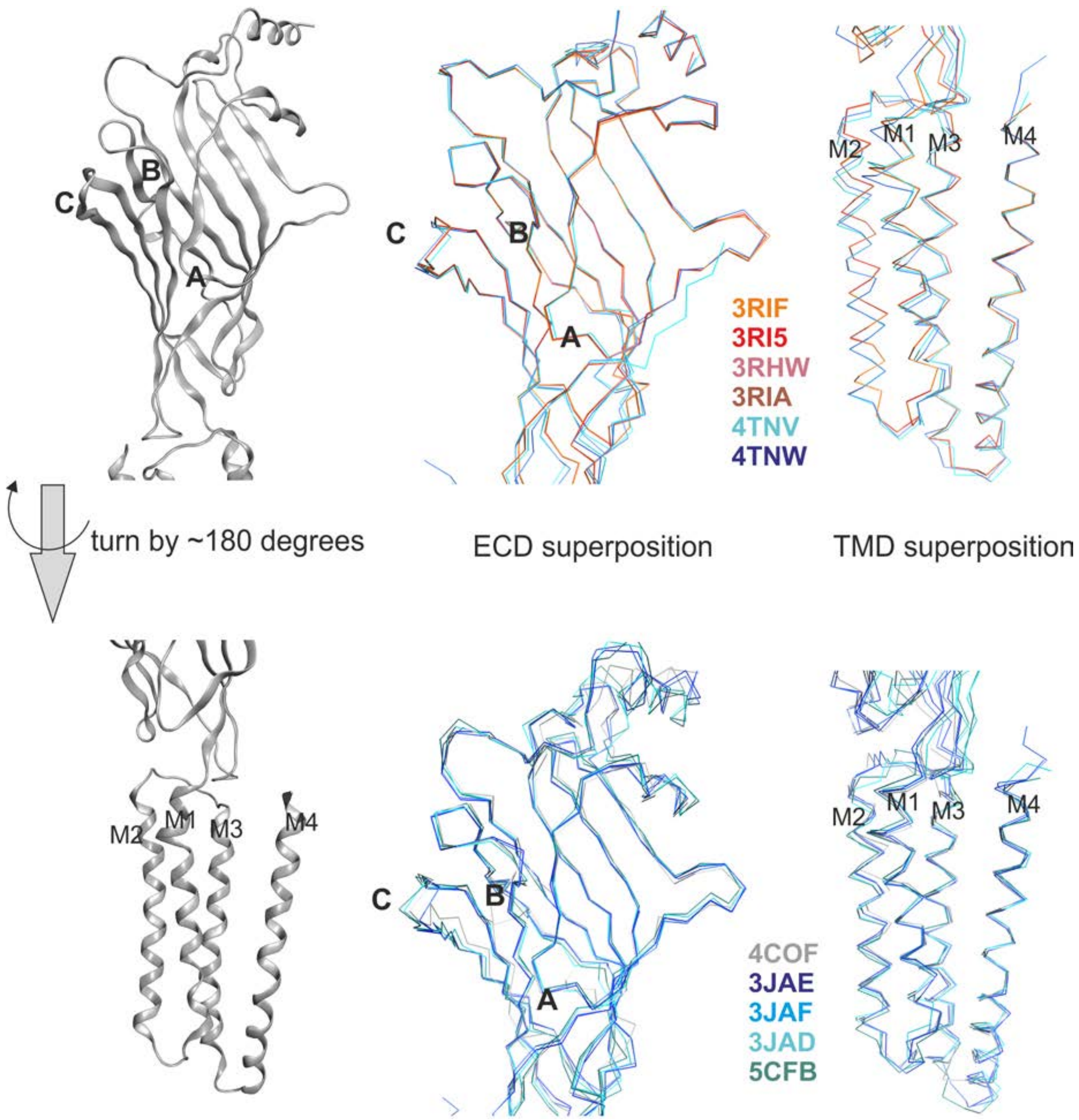

Supplementary Figure 7b: The left two ribbon images show 4COF and provide the perspective from which the images were rendered. The ECD is viewed looking “into” the plus side, so that the loop C tip is on the left edge of the image. The minus side forming β strands are therefore seen from “behind”. The subunit is turned around the longitudinal axis by ~ 180 degrees to render the TMD with the M1 helix “in front”. Of the trace renderings, the top panels show ECD and TMD superpositions respectively of single GluCl subunits from the same perspectives. Note the very high degree of structural overlap in the ECD. 3RIF, 3RIA and 3RHW are so similar to each other that their trace renderings cannot be distinguished. Note that loop C is nearly unchanged in all GluCl subunits. The bottom panel shows ECD and TMD superpositions of 4COF with all currently available GlyR structures. Note that the strychnine bound structures 3JAD and 5CFB feature a slightly more extended loop C, along with a complex reorganization of the entire ECD including a shift in the position of the N-terminal helix. Overall, both the ECD and the TMD are remarkably conserved, and nearly rigid – conformational changes are mainly changes of the relative position of the domains with respect to one another, see also Supplementary Figure 6a.

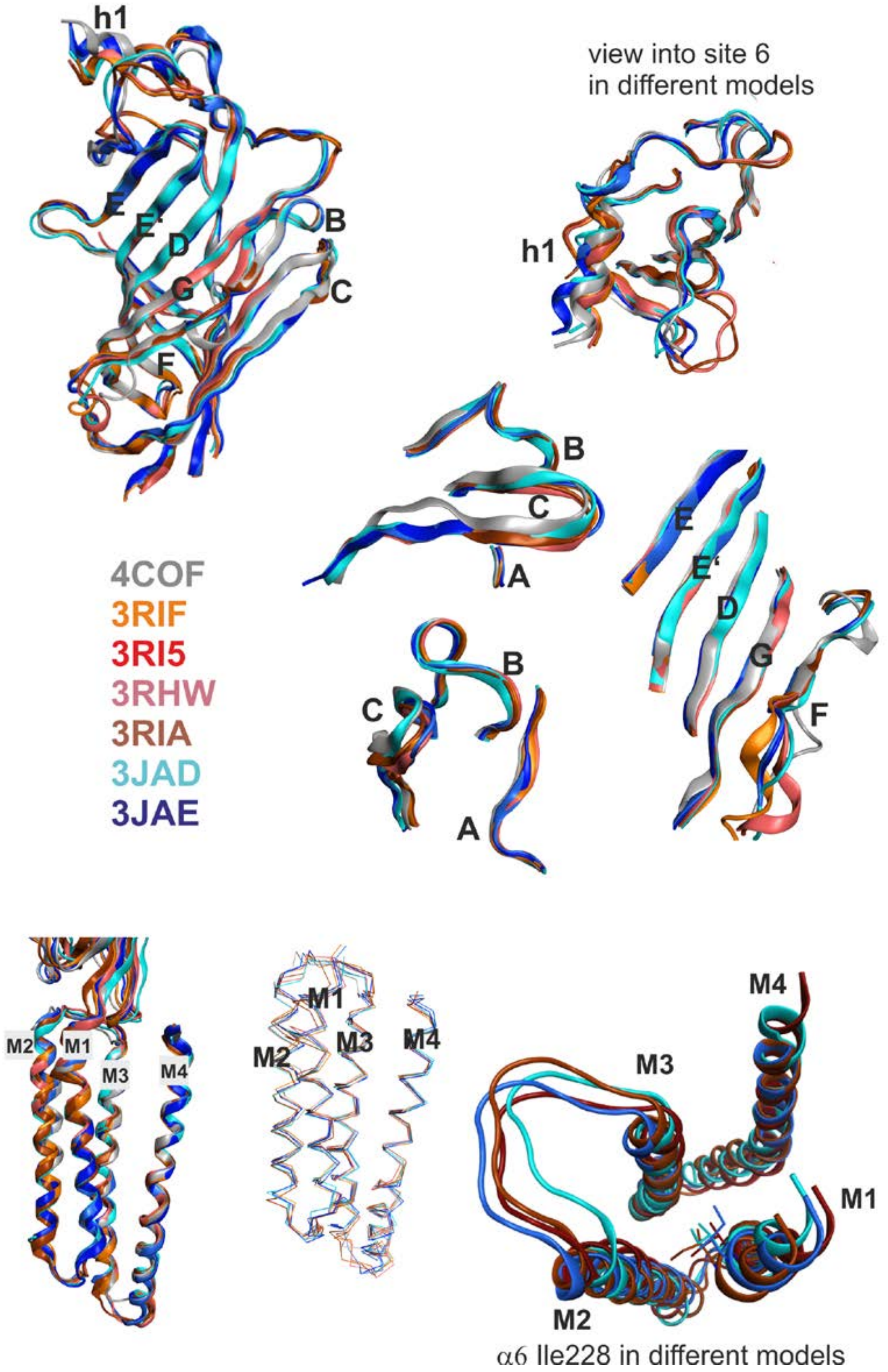

Supplementary Figure 7c: ECD and TMD of single subunits of 4COF display overall high similarity with all single subunits of GlyR and GluCl structures. The color codes of the PDB identifiers match the color codes of the ribbon and trace representations. Individual panels show the whole ECD, site 6, the extracellular interface forming loops A-C of the plus side, extracellular interface forming loops D-G of the minus side, the TMD, and the position of α6 Ile228 in different models.

8: Impact of protein motion on ECD interface geometry:

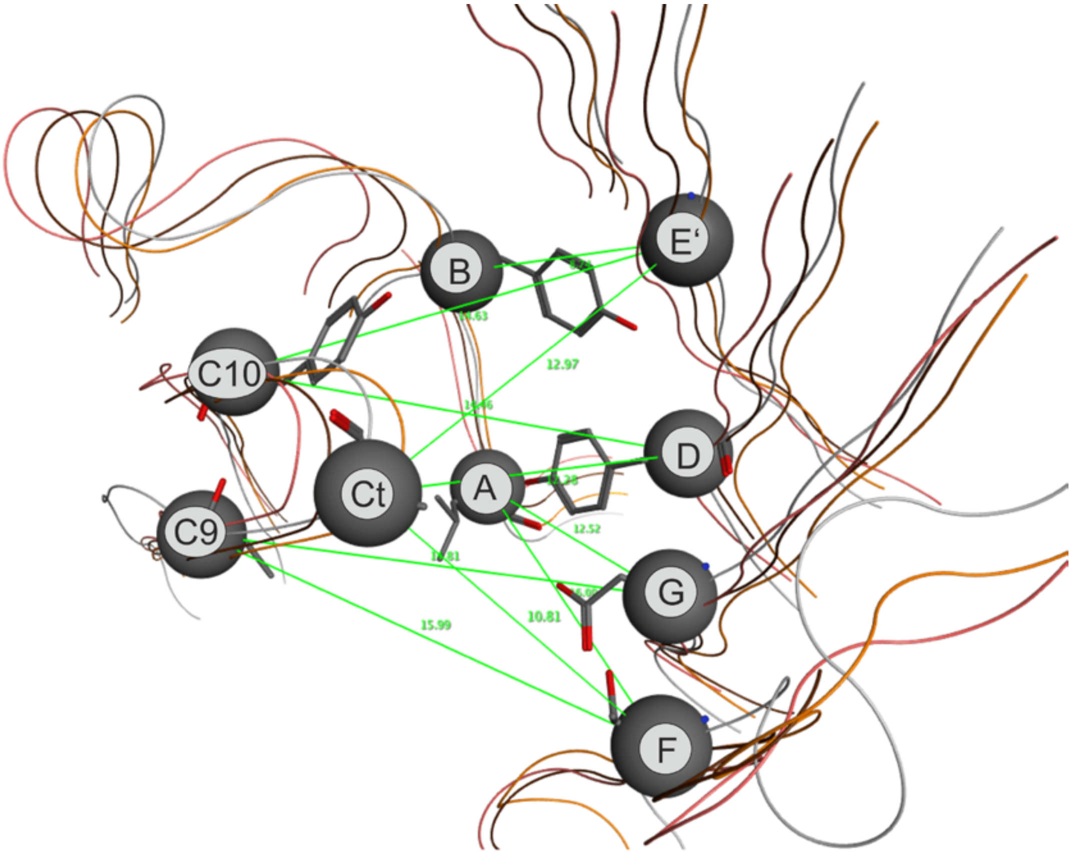

	4COF	3RIF	3RIA	3RI5	3RHW	4TNV	4TNW	3JAE	3JAF	3JAD	5CFB
**Ct-E**	12,9	11,3	11,3	11,2	11,2	11,3	12,2	**11,2**	**11,2**	**14,0**	13,9
**Ct-D**	12,2	11,8	12,1	12,0	12,0	12,3	13,0	**10,9**	11,1	14,3	**15,1**
**Ct-F**	10,6	10,3	10,4	10,5	10,5	12,1	11,2	**9,2**	9,2	12,8	**14,1**
**B-E'**	**8,8**	9,2	9,5	9,3	9,3	**10,9**	9,7	9,1	9,3	10,4	9,9
**C10-E'**	14,7	14,1	14,0	14,1	14,1	15,3	14,7	**13,7**	**13,7**	**15,4**	14,9
**C10-D**	14,6	14,9	15,2	15,2	15,3	**16,4**	15,7	**13,8**	13,9	15,3	15,6
**C9-G**	15,0	14,8	15,0	15,0	15,1	**16,5**	15,7	**12,8**	13,0	13,8	13,6
**C9-F**	16,1	15,3	15,3	15,4	15,5	**17,2**	15,7	**13,7**	13,9	15,0	14,9
**A-G**	12,7	12,8	12,7	12,9	12,9	**13,8**	12,8	12,7	12,6	12,3	**12,3**
**A-F**	16,1	16,0	15,9	16,1	16,1	**16,9**	15,7	15,8	15,8	**15,5**	15,6

Figure 8: The ECD interface geometry is represented by a set of distances between α-carbons of residues that are interface lining, and localized on the tip of loop C (Ct), at the base of loop C on strands 9 (C9) and 10 (C10) respectively, on loops A, B, D, E’, G and F. The distances for the indicated templates have been tabulated, the smallest values of each distance are highlighted in light green, the largest ones in yellow.

9: Impact of protein motion on TMD interface geometry:

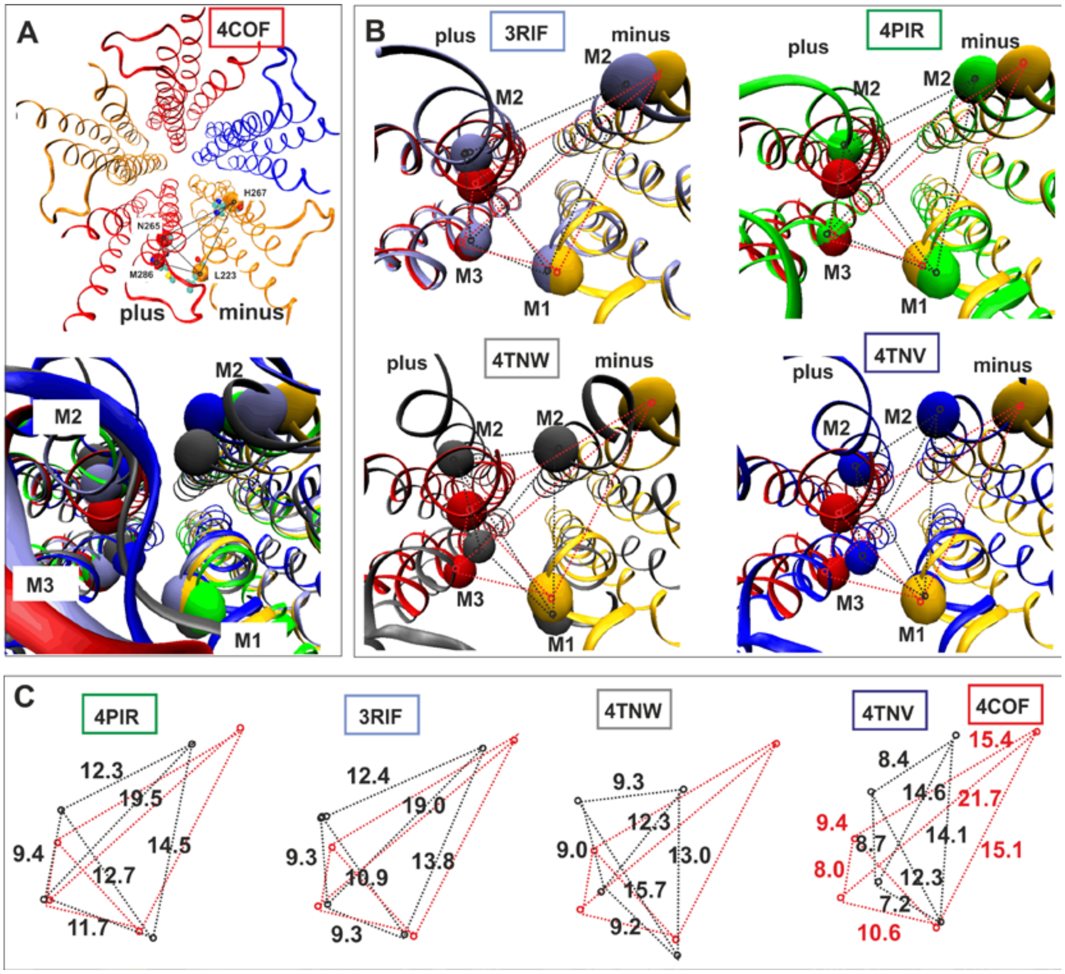

Supplementary Figure 9a. The impact of different conformations on the geometry of the TMD interface pocket. The TMD interface geometry is represented by a set of distances between α-carbons of four residues that are (at least in some conformations) interface lining and may form a pocket together. All indicated structures have been superposed by secondary structure matching (PDBeFold) as complete pentamers. **A:** Overview, the top image shows the TMD of 4COF with a color scheme indicating heteromeric assemblies modeled on this structure. Four amino acids that presumably participate in binding of either etomidate or propofol at one of the interfaces are shown in licorice, from the plus subunit β3N265 (15’, M2) and β3M286 (36’ M3); from the minus side β3L223 (-27’, M1) and β3H267 (17’, M2). The bottom image shows their α–carbons in the superposed structures in a perspective slightly tilted compared to the pentamer view. **B:** The six distances between these positions are compared pairwise to 4COF and are depicted from the same perspective as in the lower part of panel **A**. The red dashed lines always connect the α–carbons of 4COF, the black dashed lines those of the respective 3RIF, 4PIR, 4TNW and 4TNV structures. Panel **C** provides the distances.

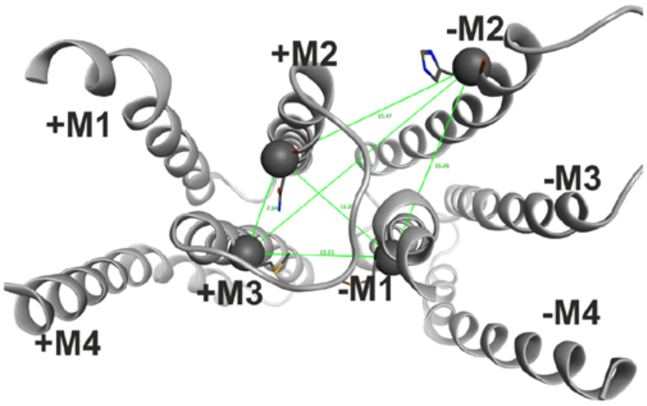

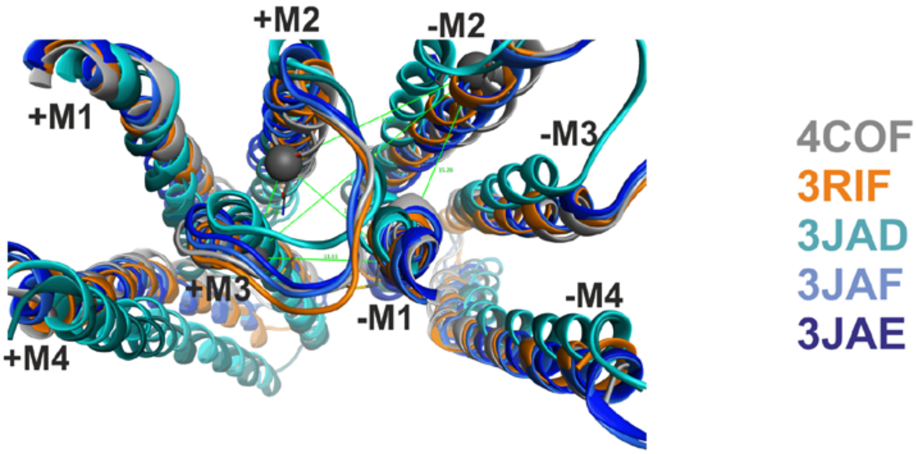

in 4COF		4COF	3JAE	3JAF	3JAD	5CFB	3RIF
N265-M286	M2+/M3+	8,0	8,6	8,7	8,1	8,9	9,3
M286-M227	M3+/M1-	11,1	11,2	10,7	8,4	8,0	9,9
M227-H267	M1-/M2-	15,3	14,2	13,9	14,4	14,1	15,0
H267-N265	M2-/M2+	15,5	13,4	13,5	8,8	9,2	12,2
N265-M227	M2+/M1-	11,4	13,6	12,6	12,3	12,6	13,2
M286-H267	M3+/M2-	21,7	19,1	19,5	15,0	15,4	18,7

Supplementary Figure 9b: A similar analysis as the one shown in 8a was performed for the GlyR structures after their release. A different residue was chosen for the M1 minus side, as indicated in the table and in the figure. The distances are tabulated below the image. The smallest distance in each row is marked light green; the largest distance in each row is marked light purple.

In heteromeric $GABA_A$ receptors, ligands interact with some degree of selectivity with the homologous TMD-interface pockets: etomidate (β+/α–), barbiturates (γ+/β–, α+/β–), alcohols, lipids, avermectin and many other compounds have been shown or proposed to use such pockets, see main text. The large conformational changes of the pocket 7 region can move side chains between interface- and non-interface positions.
